# A systematic review of dietary, nutritional, and physical activity interventions for the prevention of prostate cancer progression and mortality

**DOI:** 10.1007/s10552-015-0659-4

**Published:** 2015-09-09

**Authors:** Lucy E. Hackshaw-McGeagh, Rachel E. Perry, Verity A. Leach, Sara Qandil, Mona Jeffreys, Richard M. Martin, J. Athene Lane

**Affiliations:** National Institute for Health Research (NIHR) Bristol Nutritional Biomedical Research Unit, University Hospitals Bristol NHS Foundation Trust and the University of Bristol, Bristol, BS2 8AE UK; School of Social and Community Medicine, University of Bristol, Bristol, BS8 2PS UK; Collaborative Leadership for Applied Health Research and Care (CLAHRC) West, University Hospitals Bristol NHS Foundation Trust and the University of Bristol, Bristol, BS1 3NU UK

**Keywords:** Physical activity, Diet, Nutrition, Randomized controlled trials, Prostate cancer, Systematic review

## Abstract

**Purpose:**

Given the long-term, although potentially fatal, nature of prostate cancer, there is increasing observational evidence for the reduction in disease progression and mortality through changes in lifestyle factors.

**Methods:**

We systematically reviewed dietary, nutritional, and physical activity randomized interventions aimed at modifying prostate cancer progression and disease-specific mortality, including a detailed assessment of risk of bias and methodological quality.

**Results:**

Forty-four randomized controlled trials of lifestyle interventions, with prostate cancer progression or mortality outcomes, were identified. Substantial heterogeneity of the data prevented a meta-analysis. The included trials involved 3,418 prostate cancer patients, median 64 men per trial, from 13 countries. A trial of a nutritional supplement of pomegranate seed, green tea, broccoli, and turmeric; a trial comparing flaxseed, low-fat diet, flaxseed, and low-fat diet versus usual diet; and a trial supplementing soy, lycopene, selenium, and coenzyme Q10, all demonstrated beneficial effects. These trials were also assessed as having low risk of bias and high methodological quality (as were seven other trials with no evidence of benefit). The remaining trials were either underpowered, at high or unclear risk of bias, inadequately reported, of short duration or measured surrogate outcomes of unproven relationship to mortality or disease progression, which precluded any benefits reported being reliable.

**Conclusion:**

Large, well-designed randomized trials with clinical endpoints are recommended for lifestyle modification interventions.

**Electronic supplementary material:**

The online version of this article (doi:10.1007/s10552-015-0659-4) contains supplementary material, which is available to authorized users.

## Introduction

Prostate cancer is the most common cancer in men in the Western world [1]. In the UK, for example, it accounts for a quarter of newly diagnosed cancers [2] and one in eight men will receive a prostate cancer diagnosis [3]. Prostate cancer is often localized and grows slowly, so men may live for many years with the disease. However, prostate cancer may behave more aggressively and is an important cause of morbidity and mortality [4]. Given the long-term chronic, but potentially fatal, nature of the disease, there is growing interest in low-toxicity interventions in the tertiary prevention of morbidity and mortality due to prostate cancer. This is of particular importance as noninvasive active surveillance, as treatment for localized disease, becomes more widely implemented and increases in popularity as a strategy for reducing potential overtreatment [5]. As the number of cancer survivors in the USA increases beyond 13 million [6], the American Society of Clinical Oncology highlights the need for clinician and survivor to understand secondary prevention and lifestyle modifications that could benefit their prostate, as well as overall, health [7]. Observationally, poor diet, low levels of physical activity, and obesity are thought to play an important role in cancer, including the progression of prostate cancer [8–13]. Higher levels of physical activity have been associated with reduced rates of overall, and prostate cancer-specific, mortality [14]. World Cancer Research Fund International guidelines for cancer prevention include being physically active for at least 30 min every day, limiting consumption of energy-dense foods, eating a variety of vegetables, fruits, wholegrains, and pulses, and limiting consumption of red and processed meats [9]. Published systematic reviews in the field have tended to examine only one specific nutritional element, such as soy isoflavones [15], or have not always focused specifically on prostate cancer [16, 17]. Those that explored the implications of diet and nutrition more broadly often looked at risk of disease development, not progression and mortality [18], or did not include physical activity interventions [18, 19]. Systematic reviews with a focus on physical activity failed to include diet and nutrition interventions or restrict the population to prostate cancer patients [20]. Where diet, nutrition, and physical activity interventions have been reviewed, primary outcomes were not progression or mortality, but measures such as body weight [21], or all cancers and pre-invasive lesions were included [22]. Additionally, some reviews have not focused purely on randomized controlled trials (RCTs), which introduces further potential for bias [18], and study methodology and risk of bias were not always assessed [16, 19].

We therefore conducted a systematic review of dietary, nutritional, and physical activity interventions aimed at modifying prostate cancer progression and mortality in men with prostate cancer. We update and broaden the scope of previous systematic reviews [15–22] and undertake detailed assessment of risk of bias and methodological quality.

## Materials and methods

### Search strategy

Studies were identified through a systematic search of the following bibliographic databases from inception to July 2014: AMED, CINCH, the Cochrane library, Embase, MEDLINE, and Web of Science. The search strategy specified terms for RCTs, prostate cancer, dietary, nutritional, or physical activity interventions, and surrogate or clinical measures of prostate cancer progression or mortality (see Supplemental Data for Medline search strategy, Online Resource 1). Reference lists of all eligible full-text articles and all relevant systematic reviews that were identified were hand searched for additional studies.

### Inclusion and exclusion criteria

To be eligible, studies had to be RCTs in men with prostate cancer who were randomized to dietary, nutritional, or physical activity interventions, which reported on surrogate or clinical measures of prostate cancer progression or mortality. Dietary or nutritional interventions were considered to be those that altered the intake of foods or dietary constituents either directly (e.g., by giving vitamin supplements) or indirectly (e.g., through nutrition education). Physical activity interventions were those involving any movement using skeletal muscles. RCTs that involved a combination of dietary, nutritional, and physical activity interventions were included. Outcomes were post-intervention effects on recognized surrogate measures of prostate cancer progression [Gleason score; prostate-specific antigen (PSA)] and clinical measures of prostate cancer progression (metastases, recurrence, disease-free survival, or prostate cancer mortality). An additional outcome was circulating insulin-like growth factor (IGF). We extracted data on any adverse events that were reported. There were no language restrictions. Commentaries and other related documents were excluded unless they provided additional data.

### Data screening

All titles and abstracts were independently screened by two of three reviewers (LHM, RP, and VL) using pre-defined inclusion and exclusion criteria. Exact duplicates were removed. Any abstracts meeting the inclusion criteria were retrieved as a full article. These were then independently considered for inclusion by two of the three reviewers (LHM, RP, and VL). Any disagreements were resolved through discussion, and if necessary, the third reviewer was consulted. An additional 5 % of titles and abstracts were triple-screened for accuracy (LHM, VL, and SQ).

### Data extraction

All data were extracted by one reviewer (VL) and double-extracted by a second (LHM or RP) using a specifically designed data extraction form. Any disagreement was resolved by consulting the third reviewer. All extracted data were then checked for a final time by the third reviewer (LHM or RP). We extracted data on study characteristics, methodological quality (based on seven design and implementation questions), variables required for a Cochrane risk of bias assessment [23], and our pre-specified primary and secondary outcomes. The quality criteria assessed were: similarity of baseline characteristics and prognostic indicators between randomized arms; reporting of a power calculation and whether this sample size was achieved; reporting of withdrawal numbers and reasons by group; description of equal therapeutic time between groups. The risks of bias criteria assessed were: reporting of sequence generation; allocation concealment; blinding of participants, personnel, and outcome assessors; completeness of outcome data; and selective outcome reporting. Descriptions of what classifies as high and low risk can be found in Supplementary Table 1 (Online resource 2). Published protocols and trial registries were additionally searched, where available and when necessary, for further methodological detail related to methodological quality and risk of bias assessment. Authors were contacted if further data were required. Authors of non-peer-reviewed documents (such as conference abstracts) were contacted for published peer reviewed data; where none were provided, these were not included in the main analysis, but the description of the study included at the end of Tables 1 and 4.

**Table 1:** Characteristics of included papers

Author, country of data collection	Intervention and duration (weeks)	Total *n* intervention: control at randomization (number analyzed, if different)	Population age mean (SD)	Clinical characteristics PSA, mean (SD) Gleason (where reported)	Attrition rate (%) and recruitment rate (%)	Compliance or adherence	Total *n* of withdrawals Intervention: control
*Nutritional or dietary intervention (single factor)*							
Chen et al. [69] China	Qilan (astragalus, fenugreek, gynostremma pentaphyllan, smilaz glabra) 4	72 34:31 (72 randomized, but only data on 65)	I: 74.22 (5.94) C: 73.71 (5.25)	PSA, I: 15.76 (11.22), C: 14.98 (11.66)	10 NR	NR	7
Stenner-Liewen et al. [78] Switzerland	Pomegranate juice 4	97 49:48 (48:46)	I: 49 (8.6) C: 48 (8.4)	PSA, I: 60 (82), C: 90 (222) (based on 48 intervention group and 46 controls) Gleason,% <8, I: 46, C: 57, ≥8, I: 54, C: 43	9.3 95.1	94 (96 %) completed days 1–28	3 1:2
Freedland et al. [25] USA	Pomegranate extract 4	69 33:36	I: 60.03 (7.935) C: 57.09 (6.254)	PSA, I: 6.89 (3.884), C: 6.83 (4.274) *p* = 0.878 Gleason, % 6, I: 39.4, C: 55.6, 7, I: 54.5, C: 38.9, 8, I: 6.1, C: 2.8, 9, I: 0, C: 2.8 *p* = 0.363	NR	80 % compliance of pill. One had approved protocol deviation: 75 % compliance	NR
Paller et al. [26] USA	Pomegranate extract Up to 78	101 51:50 (45:47)	I: 71.8 (51–89) C: 73.5 (54–92)	PSADT, I: 15.1 (12.9), C: 14.4 (9.5) Gleason, % ≤6 or 3&4, I: 24, C: 74.5, 4&3, or ≥8, I: 24, C: 25.5	42 NR	58 %	9 5:4
Gee et al. [27] USA	Vitamin D 4	31 15:16 (Unclear)	I: 59.9 (5.8) C: 57.9 (6.2)	PSA, I: 11.7 (12.4), C: 6.8 (5.3) Gleason, mean (SD), I: 6.2 (1.32), C: 6.56 (0.88)	NR	15/16 took ≥95 % of study drug 11/15 took ≥99 % of study drug	NR
Wagner et al. [28] USA	Vitamin D3 3–8	66 21:22:23 (Unclear)	Total: 57.4 (6.8) I1: 58.9 (6.2) I2: 55.9 (7.3) I3: 57.6 (6.7)	PSA, Total: 6.99 (4.56), I1: 7.08 (4.55), I2: 7.02 (4.75), I3: 6.87 (4.59) Gleason, % 6, Total: 52, I1: 50, I2: 42.9, I3: 60.9, 7, Total: 48, I1: 50, I2: 57.1, I3: 39.1	4.6 70.2	97 % compliance to vitamin D3 treatment	4 I1, 2: I2, 1: I3, 1
Margalit et al. [31] USA	Beta carotene NR	383 192:191	Median (IQR) I: 73 (68–76) C: 73 (69–76)	PSA, median (IQR), I: 7.1 (5.6–13), C: 8.2 (5.5–15.8) Gleason, % 2–6, I: 67, C: 55, 7, I: 22, C: 27, 8–10, I: 11, C: 16, Missing, I: 1, C: 2	NR	I1: 80 %, C: 80 %	NR
Nguyen et al. [32] USA	Polyphenon E 3–6	50 25:25 (24:22)	I: 63.4 (5.9) C: 61.3. (5.7)	PSA, I: 6.71, (4.04), C: 7.90 (5.54) Gleason, % 6 (3&3), I: 70.8, C: 70.8, 7 (3&4), I: 12.5, C: 12.5, 7 (4&3), I: 4.2, C: 8.3, ≥8, I: 12.5, C: 8.3	4 NR	NR	2 1:1
Lazarevic et al. [75] Norway	Genistein 3–6	47	NR	NR	NR	NR	NR
Lazarevic et al. [76] Norway	Genistein 3–6	47 23:24 (23:17)	Median (range) I: 60 (53–68) C: 59 (47–70)	PSA (CI), I: 8.9 (7.0–10.8), C: 8.2 (6.4–9.9) Gleason, % 6, I: 47.8, C: 52.9, 7, I: 47.8, C: 41.2, 8, I: 4.3, C: 5.9	14.8 87	Returned pills, mean (95 % CI) I: 98.3 (97.2–99.4) C: 96.6 (93.8–99.4) *p* = 0.200	7 0:7
Paller et al. [34] (Abstract only) USA	Pomegranate extract Up to 78	104	Median, Total: 74.5	NR	NR	NR	NR
Higashihara et al. [74] Japan	Eicosapentanenoic acid 104	68 34: 34 (32:32)	I: 68 (5) C: 68 (7)	PSA, I: 7.8 (4.3), C: 10.2 (6.6) Gleason, mean (SD), I: 6.3 (0.9), C: 6.2 (1.4)	NR 84	NR	6 2:4
Stratton et al. [37] USA	Selenium Up to 260	140 47 (200 g): 47 (800 g): 46 (control)	I1: 73.6 (6.0) I2: 72.0 (7.5) C: 72.9 (6.5)	PSA, I1: 8.0 (7.0), I2: 8.3 (6.2), C: 7.4 (5.6) Gleason, % >4, I1: 87.23, I2: 91.49, C: 91.11	27.9 70.3	Mean % after 5 years. I1: 90, I2: 89, C: 90 *p* = 0.695	39 13:16:10
Vidlar et al. [70] Czech Republic	Selenomethionine (570 mg silymarin, 240 µg selenium) 26	37 19:18	I: 62.4 (6.4) C: 65.0 (3.9)	NR	NR	NR	0
Kumar et al. [41] USA	Lycopene 4–6	45 10:10:14:11	I1: 60.94 (7.05) I2: 57.54 (7.29) I3: 59.98 (6.46) C: 60.30 (6.54)	PSA, I1: 6.46 (2.74), I2: 5.86 (2.45), I3: 5.97 (4.0), C: 5.48 (3.38)	6.7 NR	NR	3 2:1
Kumar et al. [50] USA	Soy protein (genistein) 12	76 39:37 (29:30)	I: 72.5 (5.0) C: 37 (70.9)	PSA, I: 7.38 (5.62), C: 7.45 (5.36) Gleason, % >6, I: 100, C: 100	22.3 63.3	NR	17 8:9
Beer et al. [49] USA	Calcitriol (vitamin D) 4	37 17:20 (Unclear)	Median (range) I: 63 (54–71) C: 58 (46–72)	PSA, median (range), I: 6 (2.3–51.5); C: 5.8 (1.7–36) Gleason, % 5–6 I:64.7, C:50; 7 I: 23.5, C:30; 8–10 I:11.8, C:15	NR NR	NR	5
Ansari et al. [71] (Methods only) India	Lycopene 13	20	Total: median (range) 72 (56–90)	PSA, I: 50.10 Gleason, % 2–4, I: 60, 5–7, I: 25, 8–10, I: 15	NR	NR	NR
Ansari et al. [73] (Letter) India	Lycopene NR	NR	NR	NR	NR	NR	NR
Ansari et al. [72] India	Lycopene NR	54 27:27	NR	PSA, I: 250.7 (857.3), C: 259.7 (860.5)	NR	NR	NR
Stratton et al. [52] USA	Selenium Up to 260	157 43 (200 g):42 (800 g):45 (control)	I1: 73.27 (5.79) I2: 73.8 (6.44) C: 74.01 (5.9)	PSA, I1: 8.33 (5.67), I2: 8.48 (5.56), C: 8.14 (6.75) Gleason, 5–7, I1: 86, I2: 85.7, C: 84.4	33.1 82.2	NR	52
Bylund et al. [77] Sweden	Rye bran bread 11	23 12:11 (10:8)	I: 69.9 (5.3) C: 69.7 (5.4)	PSA, I: 15.0 (9.5), C: 13.5 (11.4)	21.7 NR	Mean grams of rye bread consumption. I: 267, C: 264	5 2:3
Kucuk et al. [53] USA	Lycopene 3	26 15:11	NR	NR	25.7 NR	NR	2
Kucuk et al. [54] USA	Lycopene 3	26 15:11	NR	NR	25.7 NR	NR	2
Kucuk et al. [55] USA	Lycopene 3	26 15:11	NR	NR	3.4 NR	NR	9
Kucuk et al. [56] USA	Lycopene 3	35 (26) 15:11 (35 randomized, data only presented for 26)	I: 62.3 (1.9) C: 62.0 (1.8)	PSA, I: 6.89 (0.81), C: 6.74 (0.88) Gleason, % ≤6, I: 73.3, C: 45.4, >6, I: 26.6, C: 54.5	2.6 NR	3.8 % had 93 % pill count 19.2 % had 55–79 %	9 Unclear by group
*Nutritional or dietary intervention (multiple factor)*							
Thomas et al. [64] UK	Supplement of pomegranate seed, green tea, broccoli and turmeric 26	203 136:67 (total 199)	Mean (range) Total: 74 (53–89) I: 71.8 C: 76.4	PSA, I: 6.5, C: 6.5 Gleason, % ≤7, I: 95, C: 88, >7, I: 5, C: 12 Mean, I: 6.5, C: 6.2	1.97 97.5	NR	4 2:2
Wright et al. [29] USA	Calorie reduced diet and <30 % energy from fat and nutritional teaching 6	19 10:9 (Unclear)	Median (range) I: 55 (40–66) C: 60 (47–73)	PSA, median (range), I: 4.4 (0.9–9.5), C: 4.8 (3.3–19) Gleason, 3 + 3, I: 80, C: 66.6, 3 + 4, I: 20, C: 33.3	NR	% change calories consumed, I: −46.6, C: −11.3 *p* = 0.03 between groups	NR
Bosland et al. [30] USA	Isoflavones (genistein, daidzein, glycetin) 104	177 87:90 (78:73)	I: 61.3 (7.2) C: 60.7 (6.6)	PSA, I: 7.13 (3.87), C: 7.71 (4.20) Gleason, mean (SD) 5, I: 1 (1), C: 0, 6, I: 19 (23), C: 18 (23), 7, I: 45 (56), C: 47 (60), 8, I: 11 (14), C: 7 (9), 9, I: 5 (6), C: 6 (8) *p* = 0.78	8.2 51.5	96 % consumed 90 % of pills supplied. Seven reported non-adherence; <50 % of packet, I: 3, C: 4	26 9:17
Aronson et al. [35] USA	Low-fat diet and fish oil supplementation Approx. 4	55 29:26 (27:21)	I: 60.5 (6.3) C: 60.4 (6.7)	PSA, I: 6.9 (4.9), C: 7.6 (5.6) Gleason, % 6, I: 59.3, C: 40, 7, I: 33.3, C: 55, 3&4, I: 25.9, C: 35, 4&3, I: 7.4, C: 25, 8–9, I: 7.4, C: 5	12.7 96.5	I: 89.5 %, C: 94.8 %	7 2:5
Aronson et al. [36] USA	Low-fat diet and soy 4	19 9:10 (9:9)	I: 63.8 (2.3) C: 64.7 (2.7)	PSA, I: 9.2 (2.7), C: 7.28 (1.5) Gleason, mean (SD), I: 6.2 (0.2), C: 6.2 (0.2)	10 95	% food consumed I1: 98.9, I2: 96.5	1 0:1
DeVere White et al. [38] USA	Supplement of genistein, daidzein and other isoflavones 26	66 36:30 (28:25)	I: 70.5 (9.3) C: 68.6 (7.3)	PSA, range (SD), I: 0.7–16.2 (3.7), C: 1.1–22.6 (4.7) Gleason, % 2–4, I: 0, C: 8, 5–6, I: 89.3, C: 8.4, 7, I: 7.1, C: 8, 8–10, I: 3.6, C: 0	19.7 NR	NR	13
Kumar et al. [39] USA	Isoflavones (genistein, daidzein, glycetin) 4 (±3 days)	44 12:11:10:11	I1: 59.9 (7.14) I2: 58.96 (6.41) I3: 58.66 (7.14) C: 60.30 (6.54)	PSA, I1: 4.88 (2.9), I2: 6.12 (2.6), I3: 5.08 (2.58), C: 5.48 (3.38)	2.3 NR	NR	1 1:0
Carmody et al. [40] USA	Weekly cooking classes 11	36 17:19 (Unclear)	Total: 69.1 (9)	PSA, Total: 2.96 (4.51)	33.3 NR	NR	5 (3:2)
Demark-Wahnefried et al. [42] USA	Low-fat and/or flaxseed-supplemented diets Average 4	161 40:40:40:41	I1: 60.2 (7.0) I2: 59.2 (8.0) I3: 59.3 (7.6) C: 58.2 (6.8)	PSA, I1: 5.2 (2.4), I2: 5.6 (5.0), I3: 6.8 (4.3), C: 5.2 (2.7) Gleason, % ≤5, I1: 5, I2: 0, I3: 5, C: 0, 6 (3,3), I1: 60, I2: 67, I3: 60, C: 66, 7 (3, 4), I1: 27, I2: 27, I3: 20, C: 22, 7 (4, 3), I1: 5, I2: 3, I3: 10, C: 7, ≥8, I1: 3, I2: 3, I3: 5, C: 5	7.5 25.8	Flaxseed, I1: 97.5 %, I3: 100 *p* = 0.001 (flaxseed) *p* = 0.905 (low-fat)	12 1:5:4:2
Parsons et al. [43] USA	Increased vegetables, wholegrains, beans/legumes and dietary education 26	43 30:13 (29:13)	Total: 64 (7.5) range 50–80	PSA, I: 7.21 (4.14), C: 6.94 (6.55), PSA median (range), I: 5.47 (3.00–17.2), C: 4.85 (1.77–23.0)	NR	NR	1 1:0
Li et al. [44] USA	Low-fat diet supplemented with soy protein 208	40 26:14	I: 60.2 (1.3) C: 63.3 (2.2)	PSA, I: 8 (0.71), C: 8.63 (1.35) Gleason, 5–6, I: 23.1, C: 21.4, 7, I: 53.8, C: 71.4, 8–9, I: 23.1, C: 7.1	37.5 NR	NR	15 7:8
Grainger et al. [45] USA	Lycopene and soy protein 4	41 20:21	Total: 70 (7)	NR	NR	Lycopene, mg/day, week 0–4, I1: 43, I2: 0 Week 4–8, I1: 40, I2: 36 Soy, g/day of soy Week 0–4, I1: 0, I2: 39 week 4–8, I1: 36, I2:39	0
Vaishampayan et al. [47] USA	Lycopene and soy Maximum 26	71 38:33	Median (range) I1: 73 (57–89) I2: 76 (50–91)	PSA, median (range), I1: 6.1 (1.1–147), I2: 6.9 (0.8–60.9)	1.41 NR	% Completed study I1: 60, I2: 48	1 1:0
Hoenjet et al. [61] The Netherlands	Supplement containing vitamins E and C, selenium, coenzyme C10 21	80 (Total 70)	Total mean (range): 73.9 (54–85)	PSA median (range), I: 11.3 (9.0–14.2), C: 12.2 (9.9–15.1)	12.5 NR	>90 % in 70 who completed study	10
Kranse et al. [62] The Netherlands	Verum supplement containing carotenoids, selenium, isoflavones 6	37 19:18 (15:17)	Total median (range): 70 (54–81)	PSA, median (range), Total: 3.24 (0.13–87.3)	16.2 44.6	Mean, ng/ml. Daidzein, I: 534, C: 8.0 Genestein, I: 1,589, C: 17	6 4:2
Schroder et al. [63] The Netherlands	Dietary supplement (including soy, lycopene, selenium, Co Q10) 10	49 24:25 (22:20)	Total: 69.8 (7.1)	PSA, Total: 3.29 (4.12)	14.2 NR	90 %	7 2:5
Oh et al. [51] USA	PC-SPES and DES NK	90 46:44 (43:42)	Median (range) I1:71.7 (48.5–91.3) I2:74.4 (43.6–90.3)	PSA, Median (range), I1: 46.7 (5.6–486.9), I2: 29.4 (8.0–2,548.8)	5.5 NR	NR	5 3:2
Dalais et al. [60] Australia	Heat-treated soy (genistein, daidzein, glycitein) and Flaxseed NK	29 (28) (Unclear)	I1: 61.7 (5.1) I2: 58.4 (4.9) C: 60.5 (5.2)	PSA, I1: 7.16 (3.23), I2: 6.31 (4.02), C: 5.81 (3.70) Gleason, mean (SD), I1: 6.5 (0.85), I2: 5.75 (0.9), C: 5.71 (1.38)	3.4 90.63	NR	1 group NR
*Physical activity interventions*							
Galvao et al. [58] Australia and New Zealand	Resistance training and aerobic training 52	100 50:50	I: 71.9 (5.6) C: 71.5 (7.2)	Gleason, <7, I: 4, C: 6, 7, I: 48, C: 54, >7, I: 48, C: 40	9 37.2	Mean (SD) number of sessions: 40 (11.9) 77 % attendance	22 14:8
Cormie et al. [59] Australia	Resistance training and aerobic training 13	63 32:31	I: 69.6 (6.5) C: 67.1 (7.5)	Gleason, mean (SD), I: 7.3 (0.8), C: 7.7 (1.2)	12.7 50	Mean (SD) number of sessions: 23.1 (2.7)	8 1:7
Segal et al. [67] Canada	Resistance training and aerobic training 26	121 40:40:41	I1: 66.4 (7.6) I2: 66.2 (6.8) C: 65.3 (7.6)	PSA, I1: 3.0 (3.3), I2: 2.5 (3.8), C: 3.9 (5.5) Gleason, mean (SD), I1: 6.7 (1.1), I2: 6.9 (0.8), C: 6.7 (0.9)	9 37.2	NR	11 7:3:1
Segal et al. [68] Canada	Resistance training 52	155 82:73 (81:73)	I: 68.2 (7.9) C: 67.7 (7.5)	PSA, I: 14.3 (9.0), C: 11.0 (11.2)	12.9 30.6	79 % attended 28 out of 36 sessions	20 8:12
*Nutritional and physical activity combined interventions*							
Bourke et al. [65] UK	Aerobic and resistance training and nutrition advice 26	100 (42:44)	I: 71 (6) C: 71 (8) Range 53–87	PSA, I: 2.7 (5.9), C: 3.3 (7.6)	32 73.5	94 % attended supervised exercise 82 % completed independent exercise	32 15:17
Hérbert et al. [33] USA	Healthy diet and aerobic exercise 26	54 29:25 (26:21)	I: 69.7 (8.8) C: 71.1 (8.1)	PSA, median (range), I: 0.87 (0.43–1.74), C: 0.71 (0.33–1.54) Gleason, % Missing, I: 19.2, C: 28.6, <5, I: 3.9, C: 0, 5–6, I: 23.1, C: 28.6, ≥7, I: 53.9, C: 42.9	13 96.4	NR	7 3:4
Bourke et al. [66] UK	Aerobic and resistance training and nutrition advice 13	50 25:25	I: 71.3 (6.4) C: 72.2 (7.7)	PSA, I: 3.3 (6.8), C: 5.0 (10.2) Gleason, mean (SD), I: 7 (1.3), C: 7 (1.1)	10 64.1	95 % attended exercise sessions 87 % completed self-directed exercise	22 20:12
Frattaroli et al. [46] USA	Vegan diet (supplemented with soy, fish oil, vitamin E, selenium, vitamin C) and aerobic exercise 104	93 44:49 (43:49)	Total: 66 (8)	PSA, % 4–10, 100 Gleason, % <7, 100	NR 51.4	I: 74 %, C: 78 % completed QoL and adhered *p* > 0.05	1 Group NR
Ornish et al. [48] USA	Vegan diet (supplemented with fish oil, selenium, soy, vitamins C and E) and aerobic exercise 52	93 44:49 (Unclear)	I: 65 (7) C: 67 (8)	PSA, I: 6.32 (1.72), C: 6.28 (1.66) Gleason, mean (SD), I: 5.7 (0.5), C: 5.7 (0.7)	9.7 51.3	I: 95 %, C: 45 % compliant after 12 months	9 3:6
Ornish et al. [57] USA	Low-fat, soy-supplemented vegan diet and exercise programs 52	93 46:47 (Unclear)	NR	NR	7.5 NR	83 % compliant at 12 months	7 1:6
*Unpublished data (not included in analysis)*							
Cipolla et al. [80] (Poster only) France	Sulphoraphane 32	81 40:41 (Unclear)	I: 68.8 (6.4) C: 70.4 (6.8)	PSA, I: 0.74 (0.64), C: 0.78 (0.68) Gleason, % <6, I: 13.1, C: 12.5, 6 (3 + 3), I: 10.5, C: 22.5, 7 (3 + 4), I: 44.7, C: 45, 7 (4 + 3), I: 26.3, C: 20, 8 (4 + 4), I: 2.6, C: 0, 8 (3 + 5), I: 2.6, C: 0	21 NR	NR	17 11:6
Nayan et al. [79] (Abstract only) NK	Lycopene 156 average	78 40:38	NR	NR	NR	NR	NR

*C* control, *CI* confidence interval, *I* intervention, *n* number, *NR* not reported, *PSADT* prostate-specific antigen doubling time, *SD* standard deviation

### Data analysis

Due to substantial heterogeneity across the studies in relation to intervention design, delivery mode and outcomes reported, formal pooling of the data by meta-analysis was not appropriate or possible. Therefore, a qualitative synthesis of all studies in a narrative format was undertaken.

The PRISMA statement was followed and adhered to [24]. The protocol was registered with PROSPERO International Prospective Register of systematic reviews, Ref: CRD42014008701.

## Results

### Descriptions of studies

**Fig. 1:**
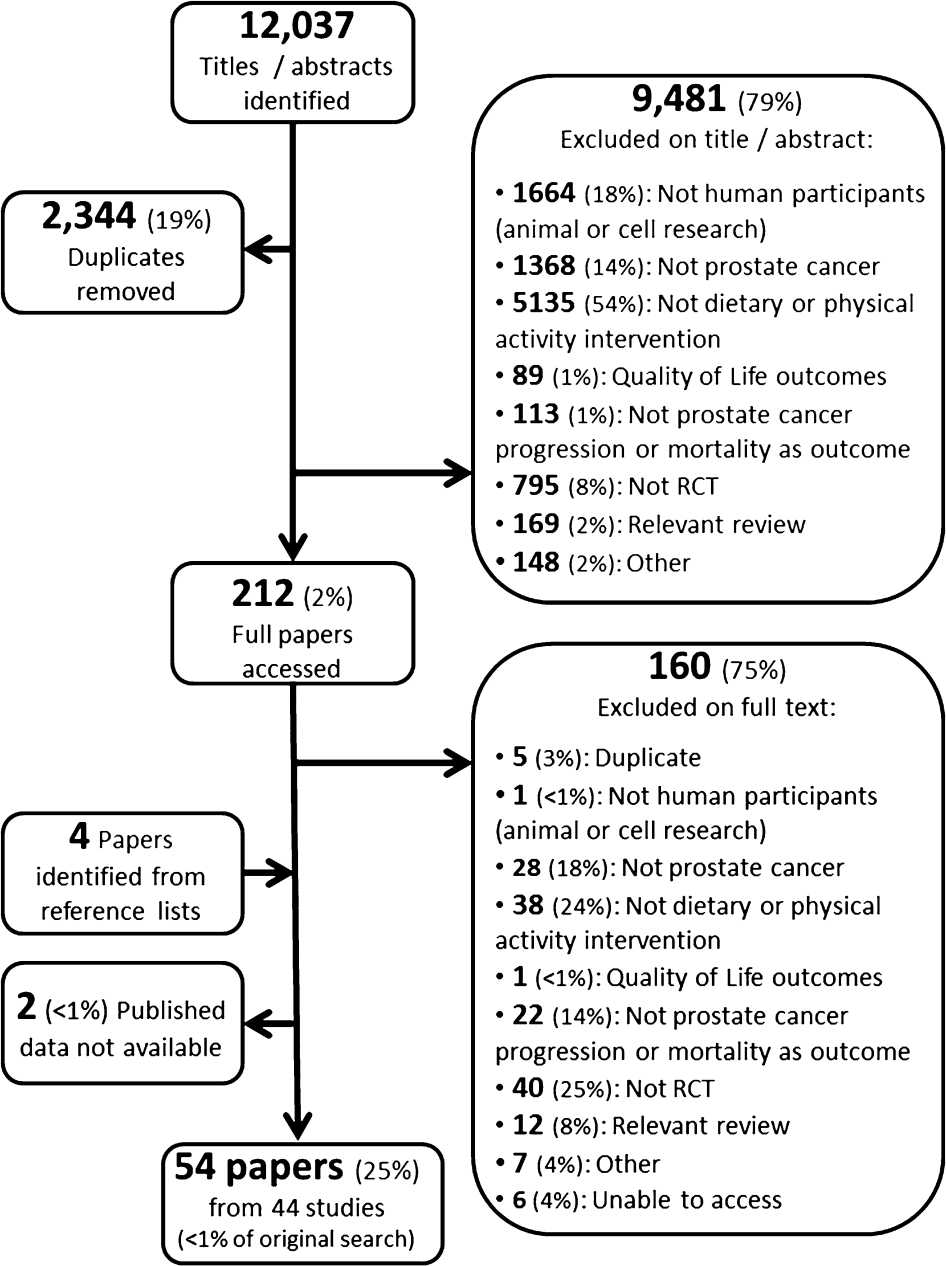
PRISMA flow diagram

The search identified 12,037 titles and abstracts, of which 9,481 (79 %) papers that did not meet our inclusion criteria and 2,344 (19 %) exact duplicates were removed. The remaining full texts of 212 (2 %) papers were retrieved and read in full; 44 RCTs reported in 54 papers met the inclusion criteria (Fig. 1).

The characteristics of the included studies are summarized in Table 1. The 44 RCTs that were eligible for inclusion in our review were published between 2001 and 2014 and involved 3,418 participants from 13 countries: 26 from the USA [25–57], three in Australia [58–60], the Netherlands [61–63], and the UK [64–66], two in Canada [67, 68], and one in each of China [69], Czech Republic [70], India [71–73], Japan [74], New Zealand [58], Norway [75, 76], Sweden [77], and Switzerland [78]. Where multiple papers were identified for the same RCT, all references are reported; however, data reported in multiple publications was only extracted once. The median size of the trials was 64 men (interquartile range 42–98, range 19–383).

The men had undergone a variety of treatments: radical prostatectomy followed by implementation of the intervention (*n* = 13 [26, 30, 33, 34, 40, 44, 45, 51, 61–63, 70, 78]) or the commencement of the intervention in men *prior* to undergoing radical prostatectomy (*n* = 13 [25, 27–29, 32, 35, 39, 41, 42, 49, 53–56, 60, 74–76]); active surveillance, active monitoring, or watchful waiting (*n* = 13 [29, 36–38, 43, 46, 48, 50–52, 57, 61, 62, 64, 77, 78]); hormone therapy or androgen deprivation therapy (ADT) (*n* = 11 [26, 34, 45, 47, 58, 59, 65–69, 78–80]); external beam radiotherapy or brachytherapy (*n* = 12 [26, 31, 33, 34, 40, 45, 51, 58, 61–63, 67, 78]); orchiectomy (*n* = 2 [69, 71–73]); chemotherapy (*n* = 1 [78]); and cryotherapy (*n* = 1 [26, 34]). The majority of studies were parallel group RCTs, with one (*n* = 31 [25, 27, 29–33, 35, 36, 38, 40, 43, 44, 46, 48–50, 53–59, 61, 64–66, 68–70, 72–78]), two (*n* = 3 [37, 52, 60, 67]), or three intervention arms (*n* = 3 [39, 41, 42]) versus usual care or some other control group. There were two dual arm parallel group RCTs without a usual care or control group comparator [26, 34, 47] and one three arm parallel group RCT without a usual care or control group comparator [28]. Four studies had a crossover design, two with one intervention arm and a usual care or control group arm [62, 63], one with two intervention arms and no usual care or control group arm [51], and one with three intervention arms and no usual care or control group arm [45].

### Excluded studies

Of the 212 texts read in full, 160 (75 %) were excluded. Thirty-eight did not involve a diet, nutrition, or physical activity intervention, and these included Ernst et al. [81], Peng et al. [82], and Sternberg et al. [83]. Twenty-two did not include prostate cancer progression or mortality as an outcome, for example James et al. [84], Zhang et al. [85], and Lee et al. [86]. Trials that only included a small proportion of prostate cancer patients within their total sample and had analyzed the data as a whole were excluded, for example Hamilton-Reeves et al. [87] (8.6 % of the study sample had prostate cancer) and Hernáandez et al. [88] (“patients with a biopsy negative for prostate cancer comprised the principal study sample” p520). Figure 1 highlights all reasons for exclusion.

### Quality of the evidence

#### Risk of bias

Overall, most of the included papers demonstrated high risk of bias on the majority of criteria or failed to adequately report how they had conducted the study on these essential criteria (Table 2). For sequence generation, half of the 44 trials were assessed as being unclear risk of bias and 22 had low risk of bias. The corresponding figures were, respectively: 30 unclear, 14 low, for allocation concealment; 22 high, five unclear, 17 low, for blinding of participants; nine high, 28 unclear, seven low, for blinding of personnel; and four high, 29 unclear, 11 low, for blinding of outcome assessor. In contrast, for completeness of outcome data 14 demonstrated high, three unclear, and 27 low risk of bias and for selective outcome reporting four demonstrated high but 40 had low risk of bias. Of note, Bosland et al. [30] and Stenner-Liewen et al. [78] were assessed to have low *overall* risk of bias, and Kucuk et al. [53–56], Beer et al. [49], Kumar et al. [50], Segal et al. [68], Demark-Wahnefried et al. [42], Schroder et al. [63], Thomas et al. [64], and Bourke et al. [66] were assessed to have relatively low *overall* risk of bias.

**Table 2:** Assessment of risk of bias

	Sequence generation	Allocation concealment	Blinding of participants	Blinding of personnel	Blinding of outcome assessor	Completeness of outcome data	Selective outcome reporting
*Nutritional interventions*							
Kucuk et al. [53–56]	+	+	−	?	+	+^a^	+
Bylund et al. [77]	?	?	+	?	+	−	+
Ansari and Gupta [71–73]	?	?	−	?	?	?	+
Beer^%^ et al. [49]	?	?	+	+	+	+	+
Kumar et al. [50]	+	+	+	+	?	−	+
Kumar et al. [41]	+	+	−	?	?	+^a^	+
Higashihara et al. [74]	?	?	−	?	?	+	+
Stratton et al. [37, 52]	+	?	+	?	?	+	−
Vidlar et al. [70]	?	?	+	?	?	+	+
Margalit et al. [31]	+	?	?	?	?	+	+
Lazarevic^%^ et al. [75, 76]	+	?	+	?	?	−	+^a,b^
Nguyen^%^ et al. [32]	?	?	+	?	?	−	−^c^
Stenner-Liewen et al. [78]	+	+	+	+	+	+	+
Chen et al. [69]	?	?	+	?	?	+	+
Wagner^%^ et al. [28]	+	?	+	?	?	+^a^	+
Gee et al. [27]	?	?	−	−	−	?	+
Freedland^%^ et al. [25]	+	?	+	?	?	+	+
Paller et al. [26, 34]	?	?	?	?	?	−	+
*Complex nutritional interventions*							
Demark-Wahnefried^%^ et al. [42]	+	+	−	−	+	+	+
Dalais et al. [60]	?	?	+	?	?	+	+
Vaishampayan et al. [47]	?	?	?	?	?	−	+
Kranse et al. [62]	?	?	+	?	?	−	+
Schroder et al. [63]	?	+	+	+	?	+^a^	+
Hoenjet et al. [61]	?	?	?	?	?	−	+
Grainger et al. [45]	?	?	−*	−	−	+	+
DeVere White^%^ et al. [38]	?	?	+	?	?	−	+
Li et al. [44]	+	?	−*	?	+^a^	?	+
Oh et al. [51]	?	?	?	?	?	+	+
Aronson^%^ et al. [36]	?	?	−*	?	?	+	+
Kumar^%^ et al. [50]	+	+	−	?	?	+	+
Carmody et al. [40]	?	?	−*	?	?	−	+
Parsons et al. [43]	?	?	−*	?	?	+	+
Aronson et al. [35]	+	?	−*	?	?	−	+
Wright et al. [29]	+	?	−*	−	?	+	+
Thomas^%^ et al. [64]	+	+	+	+	?	+	−
Bosland et al. [30]	+	+	+	+	+	−	+
*Nutritional and physical activity interventions*							
Ornish et al. [48, 51], Frattaroli et al. [46]	?	?	−*	?	+	+^a^	+
Hébert [33]	?	?	−*	?	?	−	+
Bourke et al. [66]	+	+	−*	−	+	+	+
Bourke^%^ et al. [65]	+	+	−*	−	+	−	+^a^
Physical activity interventions							
Segal et al. [68]	+	+	−*	−	+	+	+^c^
Segal^%^ et al. [67]	+	+	−*	+	?	+	−
Galvao^%^ et al. [58]	+	?	−*	−	−	+	+^c^
Cormie^%^ et al. [59]	+	+	−*	−	−	+	+^c^

Due to the nature of the interventions, we modified the Cochrane guidelines to separate blinding of participants and blinding of personnel; this addressed the difficulty in blinding participants in some of the interventions considered. Each trial was given a low (+), high (−), or unclear (?) risk of bias score for each dimension. Where a full paper was not available risk of bias was not assessed, as it was felt this would be a biased assessment without full data available

Key: + low risk of bias; − high risk of bias; ? unclear; * impossible to blind

% papers with protocols or trial registration (protocols were not accessed for the majority of studies)

^a^Not on all outcomes

^b^Information from protocol

^c^PSA reported but not pre-specified in protocol or trial registration

#### Methodological quality

The methodological quality of the trials was variable; although it was generally acceptable in the majority of the RCTs, some scored very low (Table 3). In particular, only 15 RCTs reported that they had reached an adequately powered sample size and reasons for withdrawals were described for only 20 RCTs.

**Table 3:** Methodological quality

	Similar baseline characteristics	Similar prognostic indicators	Power calculation conducted	Power sample size reached	Withdrawal numbers by gp	Withdrawal reasons by gp	Equal therapeutic time by gp
*Nutritional interventions*							
Kucuk et al. [53–56]	?	?	?	?	?	−	+
Bylund et al. [77]	−	+	−	?	+	−	+
Ansari and Gupta [71–73]	+	+	?	?	?	?	+
Beer et al. [49]	?	?	+	+	−	−	+
Kumar et al. [50]	?	?	+	−	+	−	+
Kumar et al. [41]	+	+	−	−	+	+	+
Higashihara et al. [74]	+	+	−	?	−	−	+
Stratton et al. [37, 52]	−	+	+	+	+	+	+
Vidlar et al. [70]	+	+	−	?	na	na	+
Margalit et al. [31]	+	−	?	?	−	−	+
Lazarevic et al. [75, 76]	?	?	?	?	−	−	+
Nguyen et al. [32]	+	+	−	−	+	+	+
Stenner-Liewen et al. [78]	+	+	+	+	+	−	+
Chen et al. [69]	+	+	?	?	−	−	+
Wagner et al. [28]	+	+	−	?	+	+	+
Gee et al. [27]	?	?	+	−	−	−	−
Freedland et al. [25]	+	+	+	−^a^	−	−	+
Paller et al. [26, 34]	+	+	+	+	−	−	+
*Complex nutritional interventions*							
Demark-Wahnefried et al. [42]	+	+	+	+	+	+	+
Dalais et al. [60]	+	+	−	−	−	−	+
Vaishampayan et al. [47]	?	?	?	?	+	+	+
Kranse et al. [62]	?	?	+	−	+	−	+
Schroder et al. [63]	+	+	+	−	+	+	+
Hoenjet et al. [61]	?	?	+	+	−	−	+
Grainger et al. [45]	?	?	−	?	na	na	+
DeVere White et al. [38]	+	+	+	+	−	−	+
Li et al. [44]	+	+	+	?	+	+	−
Oh et al. [51]	+	+	+	−	?	?	+
Aronson et al. [36]	+	+	+	−	+	+	+
Kumar et al. [50]	+	+	na	+	+	+	+
Carmody et al. [40]	+	+	?	na	+	+	−
Parsons et al. [43]	?	?	−	?	+	+	−
Aronson et al. [35]	?	?	−	+	+	+	+
Wright et al. [29]	−	+	−	?	−	−	−
Thomas et al. [64]	−^b^	+	?	?	+	−	+
Bosland et al. [30]	+	+	+	−	+	+	+
*Nutritional and physical activity interventions*							
Ornish et al. [48, 51], Frattaroli et al. [46]	+	+	−	−	+	+	−
Hébert [33]	+	+	+	+	+	+	−
Bourke et al. [66]	+	+	−	na	+	+	+
Bourke et al. [65]	+	+	+	+	+	+	−
*Physical activity interventions*							
Segal et al. [68]	+	+	+	+	+	−	−
Segal et al. [67]	+	+	+	+	+	−	−
Galvao et al. [58]	+	+	+	+	+	+	−
Cormie et al. [59]	+	+	+	+	+	+	−

Seven design and implementation questions were posed; RCTs were scored as yes (+), no (−), or unclear (?) for each question. Where a full paper was not available methodological quality was not assessed, as it was felt this would be a biased assessment without full data available

Key: + yes; − no; ? unclear

^a^Sample size not reached by *n* = 1

^b^Not similar on baseline age

### Interventions

The median intervention duration was 12 weeks [interquartile range 4–26 weeks (6 months), range 3–260 weeks (65 months)].

#### Single-factor dietary interventions

##### Calcitriol (vitamin D3)

The effect of calcitriol supplementation up to 2 months prior to radical prostatectomy was reported in three RCTs [27, 28, 49]. Men were randomized in the three trials, respectively, to doses of 10 µg vitamin D daily versus no supplement; 400 versus 10,000 versus 40,000 IU vitamin D3 daily; and 0.5 µg/kg calcitriol daily versus placebo. In all three trials, there was little evidence of an effect of vitamin D3 on change in total PSA, IGF-I, cell apoptosis, or proliferation.

##### Lycopene

Lycopene supplementation up to 6 weeks prior to radical prostatectomy was investigated in two RCTs reported in five publications [41, 53–56]. Men were randomized in the two trials, respectively, to doses of 15, 30, or 45 mg lycopene daily versus no supplementation and 15 mg lycopene versus usual care. No between-group differences in PSA change [41, 53–56], IGF-I change [53–56], or cellular response [41, 53–56] were observed. In a trial assessed as having high or unclear risk of bias on six of the seven criteria, where low risk of bias was only attributed to selective outcome reporting, Ansari and Gupta [71–73] randomized men, with advanced or metastatic disease, to orchiectomy alone versus orchiectomy plus 2 mg lycopene supplementation twice daily. A difference in change in PSA between the groups at 24 months was observed (*p* < 0.001); fewer intervention men had a clinically raised PSA indicating progression than the control arm (*p* < 0.05); there were fewer bone metastasis in the intervention group (*p* < 0.02), and prostate cancer mortality was lower in the intervention group (*p* < 0.001).

##### Pomegranate

Three trials [25, 26, 34, 78] randomized men to pomegranate extract supplements, one in men for four weeks prior to radical prostatectomy [25]; one for up to 18 months following radiotherapy, prostatectomy, hormone therapy or ADT, or cryotherapy [26, 34]; and one following radiotherapy, prostatectomy, hormone therapy or ADT, chemotherapy, or watchful waiting for 28 days [78]. In men randomized to 2 g of pomegranate extract daily (including 1.2 g of polyphenol), there was no difference in measures of cell proliferation, progression, or change in PSA [25]. In the studies that randomized men at a variety of TNM classification of malignant tumors stages to pomegranate extract following definitive treatment, no differences were observed in median PSA doubling time or PSA change between experimental versus control groups [26, 34]. The third trial, which was assessed to have low risk of bias, found no between-group differences in PSA change [78].

##### Genistein (soy)

The effect of genistein supplementation was investigated in two studies [50, 75, 76]. In men randomized to 30 mg genistein daily for three to six weeks prior to prostatectomy, differences in favor of the experimental, versus control, group were reported for percentage change in PSA (*p* = 0.051), cellular response (*p* = 0.033), and cell proliferation (*p* < 0.001). However, the trial was assessed as having high or unclear risk of bias on four of the seven criteria, and it was assessed to have low risk of bias for sequence generation and blinding of participants, as well as selective outcome reporting [75, 76]. Comparably, in a trial with relatively low risk of bias, in men undergoing watchful waiting, randomization to 60 mg genistein daily versus an isocaloric placebo for 12 weeks had no impact on mean change in PSA [50].

##### Selenium

Two RCTs investigated the effects of selenium [37, 52] or selenium and silymarin [70] supplementation: one in men with localized disease on active monitoring, active surveillance, or watchful waiting supplemented with 200 or 800 µg selenium versus placebo for up to 60 months; and the other in men following prostatectomy who were supplemented with selenomethionine (240 µg selenium and 570 mg silymarin) or placebo for 6 months. There were no between trial group differences in measures of PSA in either trial.

##### Other nutritional interventions

There was no evidence of any effect of any of the following interventions: Qilan capsules (consisting of astragalus, fenugreek, gynostremma, pentaphyllan, and smilaz glabra) given for 4 weeks versus placebo in men who had undergone hormone therapy or ADT and orchiectomy on PSA outcomes [69]; 50 mg of beta carotene on alternate days (for an unreported duration) versus placebo in men undergoing radiotherapy on prostate cancer mortality [31]; 800 mg of polyphenol E versus placebo daily for 3–6 weeks prior to undergoing prostatectomy on changes in PSA, IGF-I, or Gleason score or on tissue measures of cell proliferation, cell apoptosis, angiogenesis [32]; or 2.4 g eicosapentaenoic acid daily versus no intervention for 24 months in men who had undergone radical prostatectomy on PSA failure [74]. However, in men not undergoing active treatment, 295 g of rye bread versus a wheat bread control for 11 weeks resulted in increased cell apoptosis (*p* < 0.05) in the intervention group, although no effect on change in PSA or IGF-I were reported. This trial was assessed to have high or unclear risk of bias on four of seven criteria, and low risk of bias was found for blinding of participants and outcome assessors, as well as selective outcome reporting [77].

#### Multiple factor dietary interventions

##### Isoflavones

Three studies explored the effect of combinations of isoflavones within individual supplements [30, 38, 39]. In a study assessed as being of low risk of bias, Bosland et al. [30] found no effect on recurrence-free survival of powdered soy protein (combining genistein, daidzein, and glycitein) compared with calcium caseinate given for 24 months in a population of men who had undergone radical prostatectomy. Others found no effect of combinations of isoflavones on PSA measures in men due to undergo radical prostatectomy [39] or men undergoing active surveillance for a period of 6 months [38]. However, a trial assessed to have high to unclear risk of bias on four of seven criteria, where only blinding of participants, completeness of outcome data and selective outcome reporting were assessed as low risk of bias, randomising men awaiting radical prostatectomy to either 50 g of heat-treated soy, or 50 g of heat-treated soy plus 20 g of linseed, or placebo, found a difference in change in PSA between the soy-only and the placebo group (*p* = 0.02) and a difference in change in free–total PSA ratio between the two intervention groups (*p* = 0.007) and the soy-only and placebo group (*p* = 0.01) [60].

#### Other complex nutritional supplement interventions

In an RCT of men undergoing active surveillance or watchful waiting, a capsule containing pomegranate seed, green tea, broccoli and turmeric in a capsule versus placebo given for 6 months was associated with a reduced rise in PSA (*p* = 0.0008) and an increase in the percentage of participants with stable PSA at 6 months (*p* = 0.00001). This trial was reported to have relatively low risk of bias [64]. Schroder et al. [63] randomized men undergoing radiotherapy or radical prostatectomy to a supplement consisting of soy, lycopene, selenium and coenzyme Q10. The intervention was associated with improved measures of PSA during follow-up, and was assessed to have low risk of bias on five of seven criteria, however, unclear risk of bias for sequence generation and blinding of outcome assessors. Further to this, several trials of various combinations of nutrients in a variety of populations of men with prostate cancer observed no evidence for any differences [45, 47, 51, 61, 62].

##### Low-fat diet combined with other nutritional elements

Three studies combined low-fat diet with another nutritional element [35, 42, 44]. Aronson et al. [35] randomized men due to undergo radical prostatectomy to 4 weeks of a daily low-fat and fish oil diet versus a Western diet, the trial was assessed to have high or unclear risk of bias for five of seven criteria, and low risk of bias was only awarded for sequence generation and selective outcome reporting. No differences were noted in change in mean PSA or change in mean IGF-I, but there was a reduction in cell proliferation (*p* = 0.026). Li et al. [44] compared a daily low-fat, high-fiber and soy protein (40 g) diet with a standard recommended control diet given for 48 months in men who had undergone radical prostatectomy. A difference was observed in IGF-I change between the groups (*p* = 0.04); however, none was seen for change in PSA. This RCT was reported to have unclear or high risk of bias on four of seven criteria, and low risk of bias was only awarded for sequence generation, blinding of outcome assessors and selective outcome reporting. Demark-Wahnefried et al. [42] randomized men due to undergo radical prostatectomy to flaxseed, low-fat diet, or flaxseed and low-fat diet versus usual diet, over an average of 31 days. The trial was assessed to have low risk of bias for five of seven criteria, and blinding of participants and of personnel were assessed to show high risk of bias; there was a change in proliferation rate between the flaxseed only and control groups (*p* = 0.0013) but no difference between apoptotic rate, median change in PSA, or median change in IGF-I. Aronson et al. [36] randomized men undergoing active monitoring to a low-fat diet, which included 35 mg of soy protein per day for 1 month, versus a Western diet but observed no differences in mean change in PSA or mean change in IGF-I between experimental and control groups.

Three RCTs included an educational element within their complex nutritional intervention [29, 40, 43] but found no consistent effects in men awaiting radical prostatectomy or undergoing radiotherapy, active monitoring, or active surveillance on PSA or IGF-I outcomes.

#### Physical activity

Four RCTs reported a physical activity intervention, resistance and/or aerobic training [58, 59, 67, 68], but found no consistent effects in men who had undergone hormone therapy, androgen deprivation therapy, or radiotherapy on PSA-based measures of progression. One of these four trials was reported to have relatively low overall risk of bias [68].

#### Combination interventions

Four RCTS combined both a nutritional and a physical activity element in their intervention [33, 46, 48, 57, 65, 66]. All of these implemented an aerobic or aerobic and resistance training program in combination with a nutritional element. There was no consistent effect in men who had undergone radiotherapy, radical prostatectomy, active surveillance, or were on ADT. Only one of these trials had relatively low overall risk of bias [66]. Further information about all studies can be found in Table 4.

**Table 4:** Primary outcomes and summaries of included papers

Author—related publications	Intervention type and intervention duration	Prostate cancer stage (where reported) and treatment received	Systematic review outcomes (intervention vs. control only)	Outcome in original paper
*Nutritional or dietary intervention (single factor)*				
Chen et al. [69]	QiIan (astragalus, fenugreek, gynostremma, pentaphyllan, smilaz glabra) supplement, four capsules, three times a day versus P (starch)	Hormone therapy/ADT: 100 % Orchiectomy: 100 %	PSA: Change, baseline to 4 weeks; mean (SD) I: 15.76 (11.22)–3.44 (3.9), C: 14.98 (11.66)–4.16 (3.88); *p* > 0.05 between groups^d^	Unclear
Stenner-Liewen et al. [78]	Pomegranate juice, 500 ml/day, 2,294 mg/l polyphenol gallic acid versus P juice	%. T0, I: 0, C: 4. T1, I: 17, C: 17. T2, I: 17, C: 41. T3, I: 51, C: 31. T4, I: 15, C: 7, No, I: 55, C: 78. N1, I: 45, C: 22. M1, I: 44, C: 18. Watchful waiting: 28.7 % Radiotherapy: 20.2 % Prostatectomy: 15.9 % Hormone therapy/ADT: 42.5 % Radiation and prostatectomy: 15.9 % Chemotherapy: 12.7 %	PSA: Progression (phase 1 day 1–28); mean (%) I: 18 (38 %), C: 19 (41 %); *p* = 0.83^d^	Primary
			PSA: Stabilization (phase 1 day 1–28); mean (%) I: 27 (56 %), C: 26 (57 %); NS^d^	Primary
			PSA: Response (phase 1 day 1–28); >50 % mean I: 0 (0 %), C: 0 (0 %); ≥30 % mean I: 3 (6 %), C: 1 (2 %); NS^d^	Primary
Freedland et al. [25]	2,000 mg of POMx powder, including 1,200 mg polyphenol daily versus P matching pill with same administration schedule	Due to undergo prostatectomy: 100 %	PSA: Change in ratio of baseline to pre-surgery; *p* = 0.443	Secondary
			Cell proliferation; ki67 mean (SD), I: 0.60 (0.89), C: 0.76 (0.90) *p* = 0.164;	Secondary
			Nf-KB mean (SD); I: 44.44 (35.47), C: 44.85 (37.88); *p* = 0.887^d^	
			Cell development progression; ps6 kinase mean (SD); I: 46.10 (24.85),	
			C: 39.53 (26.50); *p* = 0.245	
Paller et al. [26, 34]	Pomegranate extract 1 g versus Pomegranate extract 3 g daily	Radiotherapy: 53.45 % Surgery: 51.55 % Brachytherapy: 75.2 % Hormone therapy/ADT: 27.65 % Radiation and Prostatectomy: 11.9 % Cryotherapy: 1.95 %	PSA: Doubling time, median difference; I1: 6.9 months, I2: 5.3 months; *p* = 0.554^d^	Primary
			PSA: Objective response rates; % patients I1: 2 %, I2: 2 %; *p* = NR^d^	Secondary
			Progression-free survival (stable disease); % patients I1: 78 %, I2: 82 %; *p* = NR^d^	Secondary
			Progressive disease rates; % patients I1: 20 %, I2: 16 %; *p* = NR^d^	Secondary
			PSA: Declining levels; “declining PSA seen in 13 % patients” *p* = NR Paller et al. [34]	Unclear
Gee et al. [27]	Vitamin D analog 10 µg daily versus observation	Localized: 100 % Due to undergo radical retropublic prostatectomy: 100 %	PSA: Change in total; diff between groups; Day 15; I: 8.9, C: 10; *p* = 0.397; Day 21; I: 8.3, C: 10.3; *p* = 0.024; Off study; I: 11, C: 10.5; *p* = 0.077; ITT; I: 9.9, C: 9.2; *p* = 0.156^e^	Primary
			IGF-I: Change, µg/10E6 platelets between groups; Day 15; I: 0.433, C: 0.426; *p* = 0.599; Day 21; I: 0.458, C: 0.435; *p* = 0.413; Study end; I: 0.4, C: 0.419; *p* = 0.682; ITT; I: 0.4, C: 0.418; *p* = 0.743^e^	Primary
			Change in level of intervention element, ng/ml, within intervention group; Day 8 *p* = 0.219; Day 28 *p* = 0.148^e^	Secondary
Wagner et al. [28]	Vitamin D3 doses of either 400, 10,000, or 40,000 IU/day	Localized: 100 % Due to undergo radical prostatectomy: 100 %	PSA: Change in serum, between groups; *p* = 0.60; NB. PSA was lower in the combined higher dose groups than 400 IU; *p* < 0.02^e^	Secondary
			Cell proliferation; Ki67, between groups; *p* = 0.46^e^	Primary
Margalit et al. [31]	Beta carotene (50 mg on alternate days) versus control (P)	T1/T2, I: 88, C: 85. T4/N1, I: 3, C: 3. T3, I: 7, C: 8. Missing, I: 2, C: 4 Radiotherapy: 100 %, Brachytherapy: 30.3 % External beam radiation: 78 %	Prostate cancer mortality—median FU of 10.5 years; hazard ratio = 0.85, 95 % CI (0.49–1.50) following adjustment	Primary
Nguyen et al. [32]	Polyphenon E (800 mg daily) versus P	Due to undergo surgery: 100 %	PSA: taken at 3–6 weeks; Absolute change in PSA mean (SD), ng/ml; I: −0.66 (SD 2.56), C: −0.08 (SD 1.28); *p* = 0.26; % decrease I: 58.3 %, C: 36.4 %; *p* = 0.15^d^	Secondary
			IGF-I; Absolute change mean (SD), ng/ml; I: −6.89 (20.97), C: −1.20 (21.82); *p* = 0.53 decrease %; I: 54.2 %, C: 36.4 %; *p* = 0.25^d^	Secondary
			Cell proliferation, % Ki67 mean (SD); I: 5.65 (9.47), C: 4.37 (6.11); *p* = 0.68^d^	Secondary
			Cell apoptosis, % cleaved caspase-3 mean (SD); I: 0.39 (0.57), C: 0.46 (0.64); *p* = 0.29^d^	Secondary
			Angiogenesis, *n* of microvessels mean (SD); I: 22.43 (9.93), C: 23.04 (10.40); *p* = 0.89^d^	Secondary
			Decrease in Gleason score; I: 20.8 %, C: 8.3 %; *p* = 0.22^e^	Secondary
Lazarevic et al. [75, 76]	Genistein (30 mg daily, 3–6 weeks prior to surgery) versus Control (P)	1c—I: 52.2 %, C: 76.5 % 2a—I: 47.8 %, C: 23.5 % Lazarevic et al. [76] Localized: 100 % Awaiting radical prostatectomy: 100 %	PSA; % change, mean (CI); I: −7.8 (−16.1 to 0.6); C: 4.4 (−5.0 to 13.9); *p* = 0.051; Change in mean (CI), I: 7.9 (6.6–9.2), C: 8.3 (6.5–10.2); *p* = 0.655 Lazarevic et al. [76]^d^	Primary
			Tumor response: androgen related biomarkers (KLK4); Difference in mean; *p* = 0.033	Primary
			Cell cycle regulation G3 cells; difference in expression (p27); *p* = 0.016	Primary
			Cell proliferation (Ki67) G3 cells; difference in expression; *p* < 0.001	Primary
			Cell apoptosis (BAX, BCL-2) G3 cells difference in expression; BAX *p* = 0.011; BCL-2 *p* = 0.125	Primary
			Neuroendocrine tumor response (CgA); difference in expression difference; *p* < 0.001	Primary
Higashihara et al. [74]	EPA (2.4 g/day) versus control (no intervention)	%. PT1, I: 9.38, C: 6.6. PT2, I: 68.75, C: 70. PT3>, I: 21.88, C: 23.3 Awaiting to undergo surgery—100 %	PSA: failure^a^, end of study; *n* (%) participants; I: 4 (12.5), C: 8 (26.7) *p* = 0.16^d^	Primary
Stratton et al. [37, 52]	3 arms: 200 µg/day selenium versus 800 µg/day selenium versus control (P)	Localized: 100 % Active monitoring/surveillance: 100 % (2010) Watchful waiting: 100 % (2003)	PSA: doubling time; median years; I: 6.98, I2: 8.45, C: 6.24 I1 versus control, *p* = 0.613 I2 versus control, *p* = 0.328	Primary
Vidlar et al. [70]	Selenomethionine (570 mg silymarin, 240 µg selenium) v P—Isomalt (250 mg), microcrystalline cellulose (250 mg), hydroxypropyl cellulose (10 mg)	Prostatectomy/surgery: 100 %	PSA: Median change; After 6 months PSA was unchanged in both groups	Secondary
			Median change in intervention element, selenium µmol/l; Change within both groups from baseline to 6 months *p* < 0.05	Secondary
Kumar et al. [41]	15 mg/day lycopene versus 30 mg/day lycopene versus 45 mg/day lycopene versus control (no supplement)	Localized: 100 % Due to undergo prostatectomy: 100 %	PSA: total, difference in mean; no evidence of any difference in mean *p* = 0.28 (all I arms versus C)	Primary
			Cell proliferation: Ki-67; Mean % (SD) post-intervention; I1: 2.63 (1.41), I2: 3.51 (1.43), I3: 3.64 (1.9), C: 4.22 (1.86); *p* = NR^d^	Primary
Beer et al. [49]	Calcitriol (0.5 µg/kg/day) versus control (starch)	%. T1c, I: 58.8, C: 45. T2, I: 0, C: 5. T2a, I: 29.4, C: 30. T2b, I: 5.9, C: 15. T2c, I: 0, C: 5. T3a, I: 5.9, C: 0. Due to undergo prostatectomy: 100 %	Cell apoptosis (BCL-2 and C-Myc); No BCL-2 staining in cancer cells was detected; 14 % of adenocarcinoma stained positive for C-Myc; *p* = NR^e^	Primary
			% PSA undetectable post surgically; I: 100 %; C: 84 %; *p* = NR	Secondary
Kumar et al. [50]	Soy protein (60 mg genistein daily) versus Control (standard American diet with isocaloric)	Watchful waiting: 100 %	Total PSA: Change, difference in mean (SD), baseline to 12 weeks; no evidence of any difference in mean; *p* = 0.96^d^	Primary
			Free PSA: Change, difference in mean (SD), baseline to 12 weeks; no evidence of any difference in mean; *p* = 0.13^d^	Primary
			Total testosterone: change in mean (SD); baseline to 12 weeks; no evidence of any difference in mean; *p* = 0.11^d^	Primary
			Free Testosterone: Change in mean (SD); baseline to 12 weeks; no evidence of any difference in mean; *p* = 0.15	Primary
Ansari et al. [71–73]	Orchidectomy plus lycopene (2 mg twice daily) versus orchidectomy	Advanced/metastatic: 100 % Bilateral orchiectomy and anti-androgen: 100 %	PSA: change in mean baseline, 6 and 24 months; Unclear reporting of between-group differences. Change within intervention group at 24 months; *p* < 0.001	Primary
			PSA: clinical response; % with progression; I: 7, C: 25; *p* < 0.05	Primary
			Bone metastasis; *n* (%) progression; I: 2 (7), C: 4 (15); *p* < 0.02	Primary
			Prostate cancer mortality; total death *n* (%); I: 7 (13), C: 12 22); *p* < 0.001	Primary
Bylund et al. [77]	Rye bran bread (295 g/day) versus control (wheat bread)	T2%, I: 70, C: 87.5, T3%, I: 30, C: 12.5 No active treatment: 100 %	PSA: change in mean baseline and 3 weeks; Unclear reporting of between-group differences. No changes in plasma levels of PSA were observed for total or free forms of PSA	Secondary
			IGF-I, ng/ml. Change in mean; baseline and 3 weeks; Unclear reporting of between-group differences. IGF-I remained essentially unaltered in both groups	Secondary
			Cell proliferation (Ki67, P27); Mean rate, baseline and 3 weeks; Unclear reporting of between-group differences. Within-groups Ki67 increased (*p* < 0.05) and p27 decreased (*p* < 0.05)^d^	Primary
			Cell apoptosis: TUNEL; Difference in Mean % (SD), baseline to 3 weeks; No between-group differences reported. Significant increase in intervention group I: 1.5 (1.3)–5.6 (3.1); *p* < 0.05^d^	Primary
Kucuk et al. [53–56]	Lycopene (15 mg twice daily) versus control (usual care)	Due to undergo prostatectomy: 100 %	PSA: Change, difference in mean (SE); pre to post-intervention; no evidence of any difference in mean; *p* = 0.25	Primary
			Cell apoptosis, (Bax), expression levels, mean (SE); Malignant Bax; I: 1.05 (0.29), C: 0.68 (0.18); *p* = 0.33; Benign Bax; I: 0.62 (0.1), C: 0.79 (0.11); *p* = 0.28	Primary
			Cell apoptosis (BCL-2), mean (SE); malignant BCL-2; I: 0.54 (0.01), C: 0.51 (0.06); *p* = 0.59; Benign BCL-2; I: 0.63 (0.04), C: 0.58 (0.04); *p* = 0.31	Primary
			IGF-I, difference in mean; pre to post-intervention; % change (SE); I: 28.8 (5.5), C: 29.9 (5.3) *p* = 0.88 Kucuk et al. [56]^d^. Plasma levels decreased in both groups, I: *p* = 0.0002, C: *p* = 0.0003 Kucuk et al. [53–56]^d^	Primary
*Nutritional or dietary intervention (multiple factor)*				
Thomas et al. [64]	Oral capsule containing pomegranate seed, green tea, broccoli, and turmeric versus identical P	Watchful waiting: 40 % Primary active surveillance: 60 %	PSA: Change; % rise, median (CI); I: 14.7 (3.4–36.7), C: 78.5 (48.1–115.5); *p* = 0.0008^d^	Primary
			PSA: stable % participants; After 6 months; I: 46, C: 14; *p* = 0.00001^d^	Secondary
Wright et al. [29]	Calorie reduced diet of 1,200–2,000 kcal/day and <30 % daily energy from fat. Nutritional and behavioral teaching versus continued normal diet	T1%, I: 90, C: 66.6. T2%, I: 10, C: 33.3 Active monitoring/surveillance—47 % Due to undergo prostatectomy—53 %	IGF-I: % change; baseline to 6 weeks; geometric mean. I: 17.0, C: 20.9; *p* = 0.84 between groups^e^	Primary
			Change in intervention element; Calories consumed, % change; I: −46.6, C: −11.3; *p* = 0.03 between groups^e^	Secondary
Bosland et al. [30]	Beverage powder of soy protein isolate, 19.2 g, containing, 1.24 mg genistein, 0.78 mg daidzein, 0.11 mg glycitein versus calcium caseinate, 19.8 g	T1c or T2: 100 % Prostatectomy/surgery: 100 %	Recurrence-free survival; Median time to recurrence; I: 31.5, C: 44; *p* = 0.62; % recurrence; I: 27.2, C: 29.5; HR, coefficient, 0.96 (0.53–1.72) *p* = 0.89^d^	Primary
Aronson et al. [35]	Low-fat diet and fish oil supplement (200 mg eicosapentaenoic acid and 367 mg docosahaxaenoic acid daily) versus Western diet (40 % fat, 15 % protein, 45 % carbs) (control)	Localized: 100 % Due to undergo surgery: 100 %	IGF-I: Change, mean difference; pre- and post-intervention; mean (SD), I: 8.8 (6.2), C: −0.4 (4.3); *p* = 0.25^d^	Primary
			PSA: Change, mean difference; pre- and post-intervention; mean (SD), I: 0.08 (0.4), C: −0.09 (0.3); *p* = 0.53^d^	Secondary
			Cell proliferation, % decrease of Ki67; I: 32.2 %, C: NR; *p* = 0.026^d^	Secondary
Aronson et al. [36]	Low-fat diet, 15 % kcal from fat, 30 % kcal from protein, including 35 g soy, 55 % kcal from carbohydrates, including 35 g fiber per day versus Western diet	Active monitoring: 100 %	PSA: Change at 4 weeks, mean (SD); I: 9.2 (2.7)–11.4 (5), C: 7.8 (1.5)–6.3 (3.6); *p* = 0.23^d^	Primary
			IGF-I: Change at 4 weeks, mean (SD); I: 58 (16.4), C: 24 (9); *p* = 0.09^d^	Primary
DeVere White et al. [38]	450 mg genistein, 300 mg daidzein and other isoflavones daily versus 5 g/day of inert cellulose (P)	Active monitoring/surveillance: 100 %	PSA: % change, *n* (%); Increased, I: 14 (50 %), C: 17 (68 %); >20 % increase, I: 6 (21.4 %), C: 7 (28 %); Stable/reduced, I: 14 (50 %), C: 8 (32 %); >20 % reduction, I: 3 (10.7 %), C: 1 (4 %); *p* = 0.29^d^	Primary
			Relationship of isoflavones to PSA levels. Intercept value (SE); Genistein: 0.0021 (0.0171); Daidzein: −0.0020 (0.0017); Equol: 0.01388 (0.0435); *p* = 0.25^d^	Secondary
Kumar et al. [39]	Isoflavones, 40, 60, 80 mg versus control (usual care)	Localized: 100 % Due to undergo prostatectomy: 100 %	PSA: change, difference in mean; pre to post treatment, mean (SD); I1: 4.88 (2.9)–5.52 (2.92), I2: 6.12 (2.6)–6.73 (NR), I3: 5.08 (2.58)–5.16 (8.66), C: 5.48 (3.38)–5.12 (1.86); Between-group p value = NR	Secondary
			Cell proliferation. Mean Ki67%, mean (SD); I1: 3.2 (2.25), I2: 4.11 (3.53), I3: 4.63 (2.67), C: 4.22 (1.86); *p* > 0.05, NS	Primary
Carmody et al. [40]	Dietary advice (reduced meat, dairy, increased veg, plant based diet) and cooking classes versus control (wait-list control)	Radiotherapy: 30.6 % Surgery: 55.6 % Seed implantation: 13.9 %	PSA: Kinetics, Log PSA, mean difference baseline to 11 weeks, mean (CI); I: 0.032 (0.013–0.054) to 0.011 (−0.023 to 0.047), C: 0.038 (0.018–0.057) to 0.037 (0.009–0.065); *p* = 0.28^e^	Secondary
			PSA: doubling time, mean difference in months; baseline to 3 months, mean (CI); I: 21.5 (12.8–66.8) to 58.5 (14.7–∞), C: 18.4 (12.1–39.2) to 18.7 (10.6–81); *p* = NR^e^	Secondary
Denmark-Wahnefried et al. [42]	Flaxseed-supplemented diet versus low-fat diet versus flaxseed-supplemented low-fat diet versus control (usual diet)	Due to undergo prostatectomy: 100 %	Proliferation rate: Mean Ki67; mean (CI); I1: 1.66 (1.13–2.64), I2: 2.56 (2.00–3.69), I3: 1.50 (1.05–2.65), C: 3.23 (2.42–3.92); I1 versus C: *p* = 0.0013; I2 versus C: *p* = 0.661	Primary
			Tumor apoptotic rate, *n* (%); 0 %: I1: 29 (74 %), I2: 26 (74 %), I3: 32 (89 %), C: 33 (84 %); >0–1 %: I1: 6 (16 %), I2: 5 (14 %), I3: 1 (3 %), C: 5 (13 %); >1–2 %: I1: 4 (10 %), I2: 4 (12 %), I3: 3 (8 %), C: 1 (3 %); I1 versus C: *p* = 0.880; I2 versus C: *p* = 0.730	Secondary
			PSA: Change, median difference baseline to follow up; median (CI); I1: 6.2 (4.8–7.7) to 6.4 (5–7), I2: 5.5 (4.6–6.7) to 5.6 (3.9–6.7), I3: 5.9 (4.9–9.4) to 5.7 (4.9–8.6), C: 5.3 (3.7–5.8) to 4.9 (3.5–6.2); I1 versus C: *p* = 0.286; I2 versus C: *p* = 0.764	Secondary
			IGF-I: Change, difference in median baseline to follow up, median (CI); I1: 124 (115–148) to 119 (107–133), I2: 133 (109–150) to 123 (100–141), I3: 129 (110–148) to 125 (113–139), C: 128 (106–133) to 112 (98–128); I1 versus C: *p* = 0.174; I2 versus C: *p* = 0.370	Secondary
Parsons et al. [43]	Structured dietary education and telephone based counseling. Targeted increasing intake of vegetables, wholegrains, beans/legumes versus Control, printed material with standard guidelines recommending five servings of fruit and vegetables a day	Active monitoring/surveillance: 100 %	PSA: Change, mean difference; baseline to 6 months, mean (SD); I: 7.21 (4.14) to 9.94 (12.9), C: 6.94 (6.55) to 6.88 (6.98); *p* = 0.29; PSA: change, median difference baseline to 6 months, median (range); I: 5.47 (3–17.2) to 6.39 (2.56–72.5), C: 4.85 (1.77–23) to 4.09 (1.58–24.5); *p* = 0.21^d^	Secondary
Li et al. [44]	Low-fat (15 % fat), high-fiber (18 g/1,000 kcal) with 40 g soy protein daily versus control (USDA recommender diet)	%. T1c, I: 3.8, C: 7.1. T2a, I: 7.7, C: 14.3. T2b, I: 7.7, C: 0. T2c, I: 50, C: 57.1. T3a, I: 11.5, C: 14.3. T3b, I: 7.7, C: 0. T3c, I: 11.5, C: 7.1 Surgery: 100 %	PSA: change; three participants had a raised PSA; 0.5 at 12 months; 0.7 at 18 months; 0.4 at 4 years; All other participants had PSA remaining at <0.2; *p* = NR	Secondary
			IGF: change, mean difference; baseline to 6 months, mean (SD); I: 260.4 (8.6) to 220.5 (7.9), C: 262.9 (8.6) to 259.5 (14.3); *p* = 0.04	Secondary
Grainger et al. [45]	Lycopene 25 mg/day for 4 weeks versus soy 40 g daily, versus lycopene and soy, 25 and 40 g daily	Radiotherapy (NR) Surgery (NR) Brachytherapy (NR)	PSA: Change, % with change Prolongation compared with pre-enrollment, *n* (%); I1: 13 (65 %), I2: 10 (50 %); *p* = NR; lower PSA at end of study than at enrollment, *n* (%); I1: *n* (25 %), I2: 9 (43 %); *p* = NR; NB. Outcome data given for 8 week intervention but needs to be handled with caution as intervention at 8 weeks is difficult to interpret due to being a cross over design	Secondary
			Prior to enrollment 12 men (30 %) who were in the slowest doubling time; by end of study this number had increased to 19 men (48 %); *p* = 0.08	Secondary
			IGF-I: Change; No significant changes during the course of the study for either group; *p* = NR	Secondary
Vaishampayan et al. [47]	Lycopene 15 mg twice daily versus lycopene 15 mg twice daily and soy isoflavone 40 mg twice daily	%. absence of metastases, I1: 79, I2: 70 presence of metastases, I1: 21, I2: 30 PSA progression without hormone therapy: 64.7 % Hormone therapy/ADT: 35.2 %	PSA: rate of PSA rise^b^, difference in mean. No between-group analysis reported, only reported results by treatment stratification	Primary
			PSA stabilization^c^; *n* (%) reaching stabilization; I1: 35 (95 %), I2: 22 (67 %)^d^ *p* = NR	Primary
Hoenjet et al. [61]	Supplement, vitamin C (750 mg/day), selenium (200 µg/day), vitamin E (250 mg/day), coenzyme Q10 (2 × 100 mg/day) versus control (P)	Either: CT1-4Nx Mo (with no curative treatment) or CT1-4 N + Mo Watchful waiting, radiotherapy, prostatectomy: 62.5 % No curative treatment: 37.5 %	PSA: Change, difference in mean (CI); pre to post-intervention; I: 1.3 (1.2–1.4), C: 1.1 (0.9–1.4); *p* = 0.67; NB. Geometric means are reported from nonparametric data. The outcome is presented on change in log PSA scores^d^	Primary
Kranse et al. [62]	Verum, selenium (0.6 mg daily), genestein (180 mg daily), daidzein (120 mg daily), lycopene (30 mg daily), margarine (20 mg daily) versus control (P)	% total. T1 or T2, 83, Grades 1 or 2, 60. Watchful waiting: 13.5 % Radiotherapy: 16.2 % Surgery: 70.3 %	PSA: total PSA slope; mean response; 0.024; *p* < 0.001; Treatment effect; −0.0018; *p* = 0.84^d^	Primary
			PSA: doubling time, weeks, median (CI); I: 44 (32–71), C: 41 (30–63); *p* = 0.84^d^	Primary
Schroder et al. [63]	Dietary supplement (soy (62.5 mg), lycopene (15 mg), selenium (128 mg), Co Q10 (4 mg) daily versus Control (P)	Radiotherapy: 31 % Prostatectomy/surgery: 69.4 %	PSA: slope, log2 serum total difference in median (range); I1: 0.0009 (−0.008 to 0.014); I2: 0.0022 (−0.004 to 0.014); *p* = 0.041; PSA: slope, non-transformed difference in median (range); I1: 0.0010 (−0.041 to 0.279), I2: 0.0025 (−0.003 to 0.110); *p* = 0.030^d^	Primary
			PSA: doubling time, days; I: 1,150, C: 445; Doubling time changed by factor of 2.6^d^	Primary
			Total PSA: Change concentration; Median (range); I: 0.10 (−2 to 17), C: 0.1 (−0.1 to −8); *p* = 0.076^d^	Primary
			Free PSA: I: 0 (−0.1 to 4.5), C: 0 (0–1.4); *p* = 0.988^d^	Primary
Oh et al. [51]	PC-SPES (3 capsules daily/960 mg) or DES (3 mg daily)	%. Rising PSA only, I1: 22, I2: 14. Bone metastases, I1: 41, I2: 57. Soft tissue metastases, I1: 9, I2: 11. Bone and soft tissue metastases, I1: 28, I2: 18. Radiotherapy: 18 % Surgery: 29 % Radiation and Prostatectomy: 14.5 % None: 38.9 %	PSA: decrease after 1st round of treatment, % mean (range); I1: 80 (59.3–99.4), I2: 72 (63.3–78.2); *p* = NR^e^ NB. As reported in paper	Secondary
			PSA: Time to progression; median *n* of months; I1: 5.5, I2: 2.9^d^; *p* = NR	Secondary
			PSA: Nadir after initial treatment, median (range); I1: 3.0 (0.2–16.8), I2: 22.1 (2.5–907); *p* = NR^d^	Secondary
Dalais et al. [60]	50 g HT soy or 50 g HT soy and 20 g linseed daily versus P (pearled wheat bread)	Due to undergo prostatectomy: 100 %	Total PSA: Change, difference in mean baseline to follow up, mean (SD); I1: 7.16 (3.23)–6.34 (3.05), I2: 6.31 (4.02)–6.99 (3.24), C: 5.81 (3.7)–7.11 (4.23); % change in Total PSA; I1: −12.7 %, C: 40 %; *p* = 0.02^f^; p for I2 versus C = NR	Primary
			Free PSA: Change, difference in mean baseline to follow up, mean (SD); I1: 0.69 (0.28)–0.74 (0.36), I2: 0.62 (0.26)–0.65 (0.42), C: 0.64 (0.54)–0.63 (0.48); *p* = NR^e^	Primary
			PSA: Free/total ratio; Change in total ratio; I1: 27.4 %, I2: −10 %, C: −15.6 %; I1 versus C *p* = 0.01; I1 versus I2 *p* = 0.007^e^	Primary
*Physical activity interventions*				
Galvao et al. [58]	Resistance and aerobic training versus control (education booklet)	%, T2—I: 62, C: 62, T3/T4—I: 38, C: 38 Radiotherapy/Hormone therapy/ADT: 100 %	PSA: Change, adjusted group difference in mean change at 6–12 months, mean(CI).; 6 months, 0.1 (−0.71 to 1.1); *p* = 0.687; 12 months, −0.3 (−1.4 to 0.8); *p* = 0.584	Secondary
Cormie et al. [59]	Resistance and aerobic training versus usual care	Hormone therapy/ADT: 100 %	PSA: change, adjusted group differences in mean change over 3 months, mean (CI); 0.18 (−0.25 to 0.60); *p* = 0.410	Secondary
Segal et al. [67]	Resistance training versus aerobic training versus control (usual care)	%. stage 1, I1: 0, I2: 2.5, C: 0. Stage 2, I1: 77.5, I2: 72.5, C: 85.4. Stage 3, I1: 20, I2: 22.5, C: 9.8. Stage 4, I1: 0, I2: 0, C: 2.4. Unassignable, I1: 2.5, I2: 2.5, C: 2.4 Hormone therapy/ADT: 61.2 % Radiotherapy: 100 %	PSA: Change, mean difference (CI); baseline to 24 weeks; I1: −1.75 (−3.01 to −0.51), I2: −2.14 (−3.34 to −0.94), C: −3.29 (−4.46 to −2.11); I1 versus C: 1.53 (−0.18 to 3.25); *p* = 0.09 I2 versus C: 1.14 (−0.53 to 2.82); *p* = 0.181	Secondary
Segal et al. [68]	Resistance training, 60–70 % max, 3 × per week versus waiting list	% Stage I, I: 0, C: 0. Stage II, I: 48.8, C: 47.9, Stage III, I: 13.4, C: 18.1, Stage IV, I: 20.7, C: 13.9. Unassigned, I: 17.1, C: 20.8 Scheduled to receive ADT—100 %	PSA: change; I: 1.78 decrease, C: 5.40 increase; *p* = 0.31^d^	Secondary
*Nutritional and physical activity combined interventions*				
Bourke et al. [65]	Aerobic and resistance training combined with healthy eating advice versus usual care	Locally advanced: 80 %, Advanced: 20 % Hormone therapy/ADT—100 %	PSA: change, mean at 12 weeks; I: 3.5, C: 4.6; Mean difference, unadjusted (CI): O.6 (−0.6 to 1.8); *p* = 0.35; Mean difference, adjusted (CI): 0.5 (−0.7 to 1.7); *p* = 0.41^d^	Secondary
Hébert et al. [33]	Healthy diet (decrease meat and dairy, increased veg and soy) and aerobic exercise versus control (usual care)	Radiotherapy: 36.2 % Surgery: 14.9 % Radiation and Prostatectomy: 48.9 %	PSA: Change, difference in mean baseline to 6 months, mean (CI); I: 0.87 (0.43–1.74) to 0.84 (0.42–1.68), C: 0.71 (0.33–1.54) to 0.78 (0.36–1.7); *p* = 0.45^d^	Primary
Bourke et al. [66]	Aerobic and resistance training combined with healthy diet advice versus control (usual care)	%. Advanced/metastatic, I: 24, C: 28 ADT—100 %	PSA: change, difference; baseline to 12 weeks, mean (SD); I: 3.32 (6.83)–4.55 (8.74), C: 5.02 (10.2)–6.24 (13.6); Group mean difference (CI); 0.01 (−2.2 to 2.2); greater increase in intervention group, *p* = 0.61	Secondary
			IGF-I: Change, difference; baseline to 12 weeks, mean (SD); I: 74.5 (21.5)–78.3 (22.6), C: 77.6 (25.8)–79.4 (27.2); Group mean difference (CI); 1.9 (−6.9 to 10.8); greater increase in intervention group, *p* = 0.72	Secondary
Ornish et al. [48, 57] Frattaroli et al. [46]	Aerobic exercise and vegan diet supplemented with soy (58 g), vitamin E (400 IU), selenium (200 mcg), fish oil (3 g), vitamin C (2 g) daily versus Control (usual care)	T1 or T2: 100 % Active monitoring/surveillance: 100 % Watchful waiting: 100 %	PSA: change, difference in mean baseline to 12 months, mean (SD); I: −0.25 (1.2), C: 0.38 (1.3); *p* = 0.016^d^	Primary
			PSA: Change, mean increase over 24 months, mean (SD); I: 0.88 (1.88), C: 0.99 (2.09); *p* > 0.05 Frattaroli et al. [46]^d^	Secondary
			PSA: change in velocity, ng/ml/years; I: 0.58, C: 0.50; *p* > 0.05 Frattaroli et al. [46]^d^	Secondary
			Prostate cancer treatment undergone, n; 0–12 months, I: 0, C: 6; 13–24 months; I: 2, C: 7; *p* = 0.005; Effect size (CI): 0.255 (0.053–0.437) Frattaroli et al. [46]^d^	Primary
*Unpublished data (not included in analysis)*				
Cipolla et al. [80] (Poster only)	Sulforaphane, 60 mg daily for 6 months, followed by a 2 month wash out period versus P (not stated)	No metastasis: 100 % Prostatectomy: 37 % Prostatectomy and RTE: 50 % RTE and Hormone therapy: 12.8 %	PSA: Change, ng/ml, mean (SD); I: 0.099 (0.341), C: 0.620 (1.417); *p* = 0.03^e^	Primary
Nayan et al. [79] (Abstract only)	Lycopene (8 mg daily) versus follow-up care only	Advanced/metastatic: 100 % Hormone therapy/ADT: 100 %	Disease progression; progression to hormone resistant cancer *n* (%); I: 4 (10 %), C: 18 (47.3 %); *p* = NR	Unknown

*C* control, *CI* 95 % confidence interval, *HR* hazard ratio, *I* intervention, *NR* not reported, *NS* not significant, *P* placebo, *PSA* prostate-specific antigen, *SD* standard deviation, *SE* Standard error

^a^PSA failure: PSA values were more than 0.2 ng/ml on two consecutive measurements Higashihara et al. [74]

^b^Rate of PSA rise: PSA velocity. Rate of PSA change over a period of time (http://www.upmccancercenter.com/cancer/prostate/psaelevated.cfm)

^c^PSA stabilization: for minimum of 3 months

^d^Number analyzed different to number randomized

^e^Number analyzed unclear

#### Adverse events

A variety of adverse events was reported in the included RCTs, and these most often included gastrointestinal events, such as mild abdominal pain, constipation, diarrhea and nausea; also reported were myalgia, including aches and pains and fever like symptoms, such as chills.

## Discussion

Among 54 papers reporting the results of 44 RCTs that explored dietary, nutritional, and physical activity interventions in men with prostate cancer, there was a large degree of heterogeneity with regard to intervention aims, methods of implementation and outcomes, with the quality of the research often being poor. Only three of ten studies with the lowest risk of bias and highest methodological rigor found a possible beneficial effect; a study in men undergoing watchful waiting or primary active surveillance suggested that a capsule containing pomegranate seed, green tea, broccoli and turmeric improved PSA kinetics in the intervention group compared to the control arm [64]. A study randomising men due to undergo radical prostatectomy to flaxseed, low-fat diet, or flaxseed and low-fat diet versus usual diet, over an average of 31 days, demonstrated a change in proliferation rate between the flaxseed only and control groups; however, no difference between apoptotic rate, median change in PSA, or median change in IGF-I were noted [42]. Finally, in a trial that randomized men undergoing radiotherapy or radical prostatectomy to a supplement consisting of soy, lycopene, selenium and coenzyme Q10, the intervention was associated with improved measures of PSA during follow-up. It should be noted that despite PSA being the most widely available, and cited, biomarker for prostate cancer, taken alone it may not be an appropriate surrogate marker of long-term therapeutic benefit in prostate cancer trials, which has not been proven to be a suitable replacement for a final survival endpoint [89].

Of the remaining studies with low risk of bias; an RCT randomising men, who were due to undergo radical prostatectomy, to calcitriol or control reported no difference in cell apoptosis or rise in PSA [49]. A trial where patients were randomized to 15 mg lycopene versus usual care reported no between-group differences in PSA change, IGF-I change, or cellular response. A study which scored low for all risk of bias, and also had excellent methodological quality, randomized participants to take pomegranate extract supplements following radiotherapy, prostatectomy, hormone therapy or ADT, chemotherapy, or watchful waiting for 28 days, and found no between-group differences in PSA change [78]. A complex isoflavone intervention, with strong methodological vigor and low risk of bias, resulted in no change in recurrence-free survival between groups [30], similarly, a trial, with relatively low risk of bias, assessing the effect of genistein supplementation on men undergoing watchful waiting were randomized to 60 mg genistein daily versus an isocaloric placebo for 12 weeks, no impact on mean change in PSA was reported [50]. It should be noted that these previous studies support World Cancer Research Fund International guidelines which state “Don’t use supplements to protect against cancer” [9]. One study, identified as being relatively low risk of bias, which combined aerobic and resistance training with diet advice [66], reported no difference in PSA or IGF-I outcomes. Finally, a resistance training intervention in men due to undergo ADT concluded no change in PSA [68], and this RCT was reported to have relatively low risk of bias. Most of the other studies reviewed were assessed as having high or unclear risk of bias, often with poor methodological vigor, so it is not possible to draw any conclusions from those studies.

This is the first systematic review, to our knowledge, which has combined interventions of modifiable lifestyle risk factors, with a primary outcome of prostate cancer progression or mortality. This is clinically relevant as it is unlikely that patients would make changes to a singular lifestyle behavior [90]; instead, for example, a clinician may recommend changes to diet alongside an increase in physical activity. As the number of cancer survivors living for longer increases [7], particularly for those with prostate cancer who are turning to active surveillance [5], further understanding of diet, nutritional, and physical activity interventions is of great importance.

The review was systematic and comprehensive and had no language restrictions. All papers were at least double-extracted. Risk of bias and methodological quality were assessed by at least two independent reviewers. This review does have some limitations. The primary outcomes of the review were not always reported as primary outcomes in the papers. Thus, we relied on reported secondary outcomes and the RCTs may not have been powered to detect differences in these outcomes. Meta-analysis was not possible due to heterogeneity of trial design, outcomes and statistical presentation; however, a qualitative synthesis was conducted. The limited quality of most of the RCTs, and the possibility of publication bias (which we were unable to formally assess in the absence of a meta-analysis), restricted the definitive conclusions that could be drawn.

## Conclusion

The complex nature of dietary, nutritional, and physical activity interventions, along with the slow-growing nature of prostate cancer that causes difficulties in measuring long-term clinically relevant change, makes research in this area difficult. Poor quality, variability in methodology, inconsistency of results, and a variety of proxies for prostate cancer progression make firm conclusions hard to draw. The RCTs identified in our review were generally likely to be underpowered, appeared to be at high or unclear risk of bias and were often inadequately reported, intervened for only short durations and followed-up men for surrogate outcomes of questionable relationship to clinical outcomes. Such trials are unlikely to have any clinical impact and should be abandoned in favor of large, well-designed trials with endpoints that will impact on clinical practice. These findings are in line with previous systematic reviews which concluded that the impact of interventions could not be reliably estimated due to limited, and low-quality, RCTs [18, 22].

## Electronic supplementary material

Supplementary material 1 (DOCX 14 kb)

Supplementary material 2 (DOCX 14 kb)
