# Supplementary material for: A systematic review of dietary, nutritional, and physical activity interventions for the prevention of prostate cancer progression and mortality
Source: Cancer Causes Control. 2015 Sep 9;26(11):1521–50. doi: 10.1007/s10552-015-0659-4 (PMC4596907; doi:10.1007/s10552-015-0659-4)
Supplement: Supplementary file 1 — Supplementary material 1 (DOCX 14 kb) [file 10552_2015_659_MOESM1_ESM.docx]

A systematic review of dietary, nutritional and physical activity interventions for the prevention of prostate cancer progression and mortality

Cancer Causes and Control

Lucy E. Hackshaw-McGeagh, Rachel E. Perry, Verity A. Leach, Sara Qandil, Mona Jeffreys, Richard M. Martin and J Athene Lane

Corresponding author: Dr Lucy E. Hackshaw-McGeagh, NIHR Biomedical Research Unit in Nutrition, Diet and Lifestyle and University of Bristol, lucy.hackshaw@bristol.ac.uk

**Supplemental Data (for publication only)**

Medline Search Strategy

1. prostate cancer/

2. prostat$ cancer$.ti,ab.

3. prostat$ carcinoma$.tw.

4. prostat$ carcinoma$.ti,ab.

5. prostat$ tumor$.ti,ab.

6. prostat$ tumour$.ti,ab.

7. prostat$ neoplas$.ti,ab.

8. or/1-7

9. food$.ti,ab.

10. supplement$.ti,ab.

11. diet/

12. exp diet therapy/

13. diet$.ti,ab.

14. nutri$.ti,ab.

15. dietary fat/

16. vitamin/

17. exp feeding behavior/

18. exp drinking behavior/

19. exp eating disorder/

20. nutritional disorder/

21. exp nutritional support/

22. exp appetite/

23. exp drinking/

24. exp eating/

25. exp trace element/

26. micronutrient/

27. exp plant/

28. exp fungus/

29. PSK.ti,ab.

30. krestin$.ti,ab.

31. (retinoic$ adj3 acid$).ti,ab.

32. isotretion$.ti,ab.

33. tretino$.ti,ab.

34. exp palliative therapy/

35. micronutrient$.ti,ab.

36. exp eating/

37. exp palliative therapy/

38. exp food allergy/

39. exp Dietary Carbohydrates/

40. exp Metabolic Diseases/

41. exp Antioxidants/

42. exp Calcium, Dietary/

43. exp Sodium, Dietary/

44. exp Potassium, Dietary/

45. exp Iron, Dietary/

46. exp Plants, Medicinal/

47. exp Retinoids/

48. exp Dietary Supplements/

49. exp Vitamins/

50. exp Antioxidants/

51. exp Beverages/

52. exp Nutrition Therapy/

53. exp Dietary Fats/

54. 9 or 10 or 11 or 12 or 13 or 14 or 15 or 16 or 17 or 18 or 19 or 20 or 21 or 22 or 23 or 24 or 25 or 26 or 27 or 28 or 29 or 30 or 31 or 32 or 33 or 34 or 35 or 36 or 37 or 38 or 39 or 40 or 41 or 42 or 43 or 44 or 45 or 46 or 47 or 48 or 49 or 50 or 51 or 52 or 53

55. exp Exercise Movement Techniques/

56. exp Exercise/

57. exp Physical Exertion/

58. exp Sports/

59. exercis$.ti,ab.

60. (physical$ adj5 activ$).ti,ab.

61. (physical$ adj5 fit$).ti,ab.

62. exp Dancing/

63. exp Physical Fitness/ or exp Physical Exertion/

64. exp Exercise Test/

65. exp "Physical Education and Training"/

66. exp Physical Fitness/

67. exp Exercise Movement Techniques/ or Movement/

68. exp Yoga/

69. yoga.ti,ab.

70. relaxation$.ti,ab.

71. exp Muscle Strength/

72. exp Physical Endurance/

73. 55 or 56 or 57 or 58 or 59 or 60 or 61 or 62 or 63 or 64 or 65 or 66 or 67 or 68 or 69 or 70 or 71 or 72

74. exp Randomized Controlled Trial/

75. exp Clinical Trial/

76. exp Controlled Clinical Trial/

77. RCT.ti,ab.

78. random$.ti,ab.

79. exp Random Allocation/

80. exp Double-Blind Method/

81. exp Single-Blind Method/

82. exp Placebos/ or exp Placebo Effect/

83. placebo$.ti,ab.

84. factorial$.ti,ab.

85. (crossover$ or cross over$ or cross-over$).ti,ab.

86. (doubl$ adj3 blind$).ti,ab.

87. (singl$ adj3 blind$).ti,ab.

88. assign$.ti,ab.

89. allocat$.ti,ab.

90. volunteer$.ti,ab.

91. exp Cross-Over Studies/

92. Comparative Study/

93. control$.ti,ab.

94. random$ allocation$.ti,ab.

95. allocation$ random$.ti,ab.

96. 74 or 75 or 76 or 77 or 78 or 79 or 80 or 81 or 82 or 83 or 84 or 85 or 86 or 87 or 88 or 89 or 90 or 91 or 92 or 93 or 94 or 95

97. (second$ adj5 primar$).ti,ab.

98. treatment$.ti,ab.

99. remission$.ti,ab.

100. exp treatment outcome/

101. exp disease-free survival/

102. remission induction/

103. exp remission induction/

104. exp survivors/

105. survivors/

106. exp survival analysis/

107. surviv$.ti,ab.

108. exp recurrence/

109. recur$.ti,ab.

110. mortality/

111. survival rate/

112. exp second cancer/

113. exp vital statistics/

114. neoplasm recurrence local/

115. exp prognosis/

116. (second$ adj5 cancer$).ti,ab.

117. progression.ti,ab.

118. PSA.ti,ab.

119. exp prostate specific antigen/

120. metastatic.ti,ab.

121. exp Gleason score/

122. 'T stage'.ti,ab.

123. exp Recurrence/

124. 97 or 98 or 99 or 100 or 101 or 102 or 103 or 104 or 105 or 106 or 107 or 108 or 109 or 110 or 111 or 112 or 113 or 114 or 115 or 116 or 117 or 118 or 119 or 120 or 121 or 122 or 123

125. 54 or 73

126. 8 and 125 and 96 and 124
