# Supplementary material for: A systematic review of dietary, nutritional, and physical activity interventions for the prevention of prostate cancer progression and mortality
Source: Cancer Causes Control. 2015 Sep 9;26(11):1521–50. doi: 10.1007/s10552-015-0659-4 (PMC4596907; doi:10.1007/s10552-015-0659-4)
Supplement: Supplementary file 2 — Supplementary material 2 (DOCX 14 kb) [file 10552_2015_659_MOESM2_ESM.docx]

A systematic review of dietary, nutritional and physical activity interventions for the prevention of prostate cancer progression and mortality

Cancer Causes and Control

Lucy E. Hackshaw-McGeagh, Rachel E. Perry, Verity A. Leach, Sara Qandil, Mona Jeffreys, Richard M. Martin and J Athene Lane

Corresponding author: Dr Lucy E. Hackshaw-McGeagh, NIHR Biomedical Research Unit in Nutrition, Diet and Lifestyle and University of Bristol, lucy.hackshaw@bristol.ac.uk

**Supplementary Table 1**: Risk of bias dimension descriptions for the purpose of the current review

| **Sequence generation** | *Low risk:* includes random number table, using a computer random number generator, coin tossing, shuffling cards or envelopes, throwing dice, block randomisation, drawing of lots. Simply stating ‘participants were randomised’ is not sufficient. *High risk:* includes a non-random component eg. sequence generated by odd or even date of birth, date of admission, hospital record number, allocation by judgement of the clinician, preference of the participant, results of a laboratory test or availability of the intervention. |
| --- | --- |
| **Allocation concealment** | *Low risk:* if the participants and investigators enrolling participants could not foresee assignment eg. central allocation (including telephone, web-based and pharmacy-controlled randomisation), sequentially numbered drug containers of identical appearance, sequentially numbered opaque sealed envelopes. *High risk:* if participants or investigators enrolling participants could possibly foresee assignments eg. an open random allocation schedule (e.g. a list of random numbers), assignment envelopes were used without appropriate safeguards (e.g. unsealed, non-opaque or not sequentially numbered), date of birth, case record number, or other explicitly unconcealed procedures. |
| **Blinding of participants** | *Low risk:* if blinding of participants was ensured and it was unlikely that the blinding could have been broken, participant was unable to determine which group they were in (eg. identical placebo). |
| **Blinding of personnel** | *Low risk:* if blinding of personnel (eg, those involved in running the study) was ensured and it was unlikely that the blinding could have been broken, staff were unable to determine which group the participant is in. |
| **Blinding of outcome assessors** | *Low risk:* if blinding of the outcome assessment was ensured and it was unlikely that the blinding could have been broken, person collecting outcome data was unable to determine which group the participant is in, or if there was no blinding or incomplete blinding, but the review authors judge that the outcome is not likely to be influenced by lack of blinding. For patient-reported outcomes, where the patient was the outcome assessor (e.g., pain, disability), there is low risk for outcome assessor if there is a low risk for participant blinding. For outcome criteria that are clinical or therapeutic events that will be determined by the care providers, low risk would result if the care providers were blinded to participant group. |
| **Completeness of outcome data** | *Low risk:* if all randomised participants are reported and analysed in the group they were allocated to for the most important moments of effect measurement, irrespective of noncompliance or drop out. Up to 10% loss is considered to be low risk. |
| **Selective outcome reporting** | *Low risk:* if all study pre specified outcomes have been reported, additionally, *high risk* if authors have reported on outcomes that were not pre specified. Study protocols and trial registration accessed where available. |
